# The Effects of Nutrient Signaling Regulators in Combination with Phytocannabinoids on the Senescence-Associated Phenotype in Human Dermal Fibroblasts

**DOI:** 10.3390/ijms23158804

**Published:** 2022-08-08

**Authors:** Marta Gerasymchuk, Gregory Ian Robinson, Olga Kovalchuk, Igor Kovalchuk

**Affiliations:** Department of Biological Sciences, University of Lethbridge, Lethbridge, AB T1K 3M4, Canada

**Keywords:** aging, skin, fibroblast, stress-induced premature senescence, metformin, triacetylresveratrol, rapamycin, THC, CBD

## Abstract

Identifying effective anti-aging compounds is a cornerstone of modern longevity, aging, and skin-health research. There is considerable evidence of the effectiveness of nutrient signaling regulators such as metformin, resveratrol, and rapamycin in longevity and anti-aging studies; however, their potential protective role in skin aging is controversial. In light of the increasing appearance of phytocannabinoids in beauty products without rigorous research on their rejuvenation efficacy, we decided to investigate the potential role of phytocannabinoids in combination with nutrient signaling regulators in skin rejuvenation. Utilizing CCD-1064Sk skin fibroblasts, the effect of metformin, triacetylresveratrol, and rapamycin combined with phytocannabinoids on cellular viability, functional activity, metabolic function, and nuclear architecture was tested. We found triacetylresveratrol combined with cannabidiol increased the viability of skin fibroblasts (*p* < 0.0001), restored wound-healing functional activity (*p* < 0.001), reduced metabolic dysfunction, and ameliorated nuclear eccentricity and circularity in senescent fibroblasts (*p* < 0.01). Conversely, metformin with or without phytocannabinoids did not show any beneficial effects on functional activity, while rapamycin inhibited cell viability (*p* < 0.01) and the speed of wound healing (*p* < 0.001). Therefore, triacetylresveratrol and cannabidiol can be a valuable source of biologically active substances used in aging and more studies using animals to confirm the efficacy of cannabidiol combined with triacetylresveratrol should be performed.

## 1. Introduction

The life expectancy in most developed countries almost doubled within the last century [1]. Longevity is a double-edged sword, as people live longer, life quality can be adversely affected by age-related diseases (ARDs). Research teams around the globe are focusing on investigating aging and treatment strategies to delay or prevent the development of ARDs to improve quality of life [2,3]. A significant challenge of aging research is distinguishing the pathogenesis of cell and tissue senescence from the myriad of changes that accompany it. Substantial research details the causes and mechanisms of aging; however, we still cannot stop aging, but only alleviate or delay some ARDs and their symptoms.

Few drugs and nutrients directed at the elimination of detrimental effects of aging have been discovered, including nutrient signaling regulators (NSRs) [3,4]. Nutrient signaling regulators maintain nutrient-sensitive signaling pathways and are vital for biological processes [3]. NSRs were shown to regulate processes related to age-related diseases such as organismal survival, growth, metabolism, signaling systems, including sirtuin, mammalian target of the rapamycin (mTOR), and AMP-activated protein kinase (AMPK) [5,6]. Among the most studied in this group are sirtuin regulators [7,8,9], mTOR inhibitors [10,11,12,13], and AMPK activators [1,14,15,16]. NSRs have been tested on eukaryotic organisms from yeast to humans, showing beneficial results and extending lifespans in model organisms by mainly targeting the master regulators of aging that affect autophagy, inflammation, and oxidative stress. In clinical trials, these drugs have been shown to prevent cardiovascular diseases, reduce inflammation, and potentially inhibit carcinogenesis [17]. Current studies in the field of aging are focused on the most popular anti-aging therapeutics: rapamycin, 1,1-Dimethylbiguanide (metformin), and 3,5,4′-trihydroxy-trans-stilbene (resveratrol).

Rapamycin is a central modulator of the mTOR pathway; it regulates transcription and protein synthesis by integrating with various signal transductions pathways to control growth, apoptosis, and autophagy in cells. The mTOR pathway is the crucial controller of growth in animals and is the fundamental link between the availability of nutrients in the environment and the control of most anabolic and catabolic processes [18]. Furthermore, mTOR drives growth-like conversion from quiescence to senescence in response to cell-cycle arrest [19]. Alterations in mTOR pathway regulation are commonly involved in several genetic diseases, including aging and ARDs in humans [20,21]. In addition, recent reports have shown deregulation of mTOR signaling in ARDs and aging in multiple model organisms [18,22,23].

Rapamycin and its analogs are currently approved by the United States Food and Drug Administration for the treatment of a number of conditions, as they have been demonstrated, both in vitro and in vivo, to slow aging and extend lifespan in different species [21] and prevent ARDs [19]. 

Apart from different diseases, rapamycin was also recommended in cosmetic practice for anti-aging and skin rejuvenation purposes. Rapamycin-containing cream was effective when applied to selected areas, like the hands and face, especially skin affected by age-related spots and dermatopathies [19]. Topical usage is considered safer and might spare the patients from the adverse effects of systemic treatment with rapamycin [24]; however, some evidence suggests that solely topical use of rapamycin may be insufficient to improve skin conditions [19]. Hence, use of rapamycin combined with other anti-aging compounds or natural products like phytocannabinoids for topical application might open new perspectives in the aging abatement and rejuvenation strategies.

Metformin is a widely used anti-hyperglycemic drug [25]. Mechanistically, metformin affects different cellular targets. Of those, it causes inhibition of the respiratory complex I of the electron transport chain [26,27], directly affecting reactions requiring adenosine triphosphate, and indirectly acting on the activation of AMPK [27,28]. As a result, this causes elevated accumulation of reduced nicotinamide adenine dinucleotide (NADH) compared to the oxidized form (NAD+) and inhibits production of NADH-ubiquinone oxidoreductase, which is localized on the mitochondrial membrane, thus activating AMPK and suppressing gluconeogenesis [26,29]. Subsequently, this leads to fatty acid synthesis and mTOR signaling network suppression [30], causing reduced cellular energy consumption. Moreover, studies have shown that long-term use of metformin alleviates oxidative damage and chronic inflammation, reduces cognitive deterioration, and prolongs health and life [31]. As a biguanide, metformin acts in a dietary restriction manner and on oxidative stress pathways in a lifespan-extending effect [25]. It reduces advanced glycation end-product (AGEs) accumulation in response to massive reactive oxygen species (ROS) generation in high glucose conditions. Accelerated dermal aging is directly correlated with increased protein deterioration in the existing collagen because of crosslinking. The progressive increase in AGEs affects cellular macromolecules during aging through oxidative damage and leads to activation of nuclear factor kappa-light-chain-enhancer of activated B cells (NF-κB). Metformin has been shown to protect T3 fibroblasts in vitro under high glucose conditions inducing cell proliferation, collagen I and III production, apoptosis inhibition, and reducing NF-κB (p65) activity [15]. In response to hypoglycemic conditions, metformin inhibits senescence-associated secretory phenotypes by repressing NF-κB pathway activation [32]. Thus, metformin may be a promising pharmacological dermal anti-aging remedy with a complex beneficial impact on the physiology of the skin and entire organism.

Resveratrol is a type of natural phenol and a phytoalexin that can be obtained from several sources, including red wine, grapes, and certain medicinal plants [33,34,35]. Nevertheless, resveratrol is often used as a popular dietary supplement [26]. Resveratrol’s anti-aging characteristics have been investigated in different species [9]. It can alleviate the inflammatory phenotype in senescent-induced human fibroblasts through upregulation of autophagic pathways [36]. Resveratrol is known to prevent ARDs and extend the lifespan of mice and rats through sirtuin (SIRT) 1 pathway modulation [37,38,39,40]. SIRT1 is a NAD(+)-dependent deacetylase involved in gene silencing, anti-oxidative stress, anti-apoptosis, and inhibition of inflammation [34,41]. Resveratrol can increase SIRT1 during aging and chronic inflammation, processes that are associated with reduced SIRT1 levels and activity in response to oxidative stress [3]. A recent study showed that resveratrol significantly elevates the SIRT1 activity, inhibits the NF-κB pathway, and prevents the loss of intestinal stem cells [34]. Nonetheless, resveratrol has not been shown to prolong the life expectancy of mice [42]. Additionally, Luz et al. (2012) reported that low resveratrol doses and red wine supplementation improved vascular function and aerobic capacity, reduced p53 and p16 senescence biomarkers in rats, but did not extend their lifespan either [43]. Furthermore, resveratrol demonstrates a typical dose-dependent response with a small therapeutic window at low doses, whereas at higher doses (50 or 100 μM), resveratrol can reduce cellular quantity and augment apoptosis or necrosis [44]. 

There are numerous limitations in resveratrol effectiveness towards longevity and anti-aging because of its limited bioavailability [35,44,45]. Alternatively, the acetylated analog triacetylresveratrol (TRSV) has higher bioavailability [46,47]. Due to its higher hydrophobic nature, TRSV, compared to resveratrol, has been shown to be more effective in interacting with and crossing phospholipid bilayers [48]. Moreover, findings show TRSV might be a more potent therapeutic agent than resveratrol and can affect proliferation and apoptosis by decreasing NF-κB phosphorylation [46], and attenuate inflammation via the p38 mitogen-activated protein kinase/SIRT1 pathway [49]. Conversely, no studies have tested the anti-aging or rejuvenation properties of TRSV.

Some of the beneficial effects of rapamycin, metformin, and resveratrol have been achieved through cutaneous application. It was shown that locally applied metformin and TRSV, but not rapamycin, improved epidermis wound healing and collagen deposition in rodents [50]. Recent clinical studies have supported the efficacy of resveratrol and its analogs (i.e., resveratryl triacetate and resveratryl triglycolate) in human skin lightening and anti-aging because of its anti-inflammatory, anti-proliferative, and anti-pigmentation properties [51]. 

Similar to TRSV, information on anti-aging properties of phytocannabinoids is also scarce. Recent studies showed that topical application of hydrogels based on cannabis extracts have a beneficial effect on skin hydration, and inhibit the activity of matrix metalloproteinases (MMPs), collagenase, and elastase via reduction of oxidative stress markers; the extracts were shown to inhibit skin aging processes and positively affect the viability of skin cells [52]. Martinelli et al. (2022) noted photoprotective, antioxidant, and anti-inflammatory mechanisms of phytocannabinoids, emphasizing the possible impact of CBD on cell differentiation in the skin, especially in the case of dermatological disorders like psoriasis [53]. 

We hypothesize that the combination of NSRs and phytocannabinoids (pCBs) may have a beneficial anti-aging and potential rejuvenation effect. While the notion of using metformin, rapamycin, or resveratrol for skin rejuvenation is not novel, combining them with phytocannabinoids and using TRSV instead of resveratrol is a novel approach in anti-aging and rejuvenation strategies for aging skin.

In this study, we characterize the anti-aging effects of NSRs, metformin, rapamycin, and TRSV alone or in combination with delta-9-tetrahydrocannabinol (THC) and cannabidiol (CBD) on functional assays and molecular targets involved in pathogenesis of cellular senescence. We used the stress-induced premature senescence (SIPS) model of human dermal fibroblasts, which we have previously shown to be a reliable and effective model of SIPS [54], to assess anti-aging effects of phytocannabinoids and NSRs.

## 2. Results

### 2.1. Setup of the Senescence Model and Treatments

**Figure 1:**
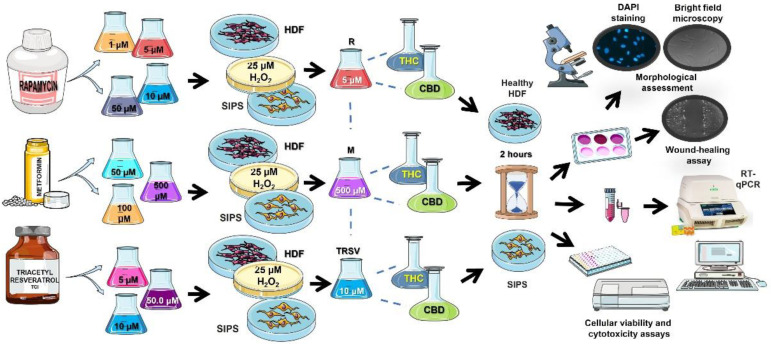
Testing of different concentrations of anti-aging drugs on prematurely senescent dermal fibroblasts. The figure shows steps to discover the efficient dose in treating senescent fibroblasts among three popular anti-aging drugs: rapamycin (R), metformin (M), and triacetylresveratrol (TRSV). CBD, cannabidiol; HDF, human dermal fibroblasts; H_2_O_2_, hydrogen peroxide; SIPS, stress-induced premature senescence; THC, Δ-9-tetrahydrocannabinol. Dashed lines show a combination of treatments. This figure was created using images from Servier Medical Art Commons Attribution 3.0 Unported License (http://smart.servier.com (accessed on 26 September 2020)).

In this study, we analyzed anti-aging effects of three NSRs, metformin, rapamycin and TRSV, in combination with THC, CBD, or THC and CBD using healthy and prematurely senescent dermal fibroblasts. The following aging-related characteristics were tested: cell senescence, cell growth rate and viability, gene expression profile, functional activity based on extracellular matrix (ECM) components production, and speed of wound healing (Figure 1).

### 2.2. Phytocannabinoids Combined with Nutrient Signaling Regulators Preserve Cellular Viability in SIPS Fibroblasts

The efficacy of NSRs was initially tested by the 3-[4,5-dimethylthiazol-2-yl]-2,5 diphenyl tetrazolium bromide (MTT) cell viability assay to detect optimal safe doses with potential anti-aging properties. MTT measures the number of viable cells via a colorimetric reaction with endogenous nicotinamide adenine dinucleotide phosphate-dependent cellular oxidoreductase enzymes reducing the tetrazolium dye.

A range of doses was initially tested for each drug (Figure S1). The MTT assay detected significant variability in treatment results depending on the dose, time of exposure, and fibroblast condition (healthy or senescent). In healthy CCD-1064Sk fibroblasts, passage 11 (p. 11), one day of exposure with 50 μM of metformin increased cellular viability, while 10 μM of TRSV showed a significantly lower cell viability (Figure S1A,E). At the same time, none of the rapamycin concentrations induced a significant effect compared to the other treatments (Figure S1I).

The viability of fibroblasts induced to be senescent via 25 µM of hydrogen peroxide (H_2_O_2_) was similarly reduced compared to healthy fibroblasts, as previously published [54]. After 1 day of exposure, THC and CBD showed the best cell growth stimulation (Figure S1F). No noticeable effects were seen for metformin or rapamycin administration (Figure S1B,J), whereas TRSV exposure decreased cell viability compared to the vehicle (Figure S1F).

Following 5 days of exposure to different concentrations of metformin, healthy CCD-1064Sk fibroblasts were not different from those exposed to the vehicle (Figure S1C). In contrast, both 5 and 10 μM of TRSV induced significantly lower cell viability compared to the untreated, but no difference was seen compared to the vehicle (Figure S1G). Surprisingly, all treatments of rapamycin induced significantly lower cell viability compared to the vehicle and untreated (Figure S1K). 

Simultaneously, the highest cell viability after the five-day exposure in the senescent fibroblasts was achieved with 50 μM of TRSV (Figure S1H), while metformin showed a trend to increase cell viability (Figure S1D) and rapamycin-treated fibroblasts demonstrated no change at all (Figure S1L). 

For the following experiments, we used 500 μM of metformin as no observable cytotoxic effects were shown (Figure S1A–D). Furthermore, this concentration was utilized in previous publications that displayed therapeutic effects on fibroblasts [55]. Similarly, 5 μM of rapamycin was shown to have therapeutic effects in human dermal fibroblasts [10], while our data showed no observable cytotoxic effects except for after 5 days, whereby all concentrations moderately inhibited cell growth in senescent cells compared to the vehicle (Figure S1K). Previous publications commonly utilized resveratrol up to doses of 50 μM [56,57]; however, triacetylresveratrol, the acetylated form of resveratrol, has a higher reported bioavailability but with a similar mechanism of action [46,47]. Although our cell viability data showed very similar results between different doses of TRSV (Figure S1E,F), San Hipólito-Luengo et al. (2017) has shown a biphasic effect of resveratrol, whereby doses of 1–10 μM increased cell viability, while doses of 50–100 μM induced apoptosis and necrosis [44]. We opted to utilize the 10 μM dose of TRSV for the following experiments to prevent apoptosis and necrosis induction. Last, we chose doses of 2 μM for both THC and CBD as these doses were well-tolerated in stromal cells but higher doses had cytotoxic effects [58]. 

**Figure 2:**
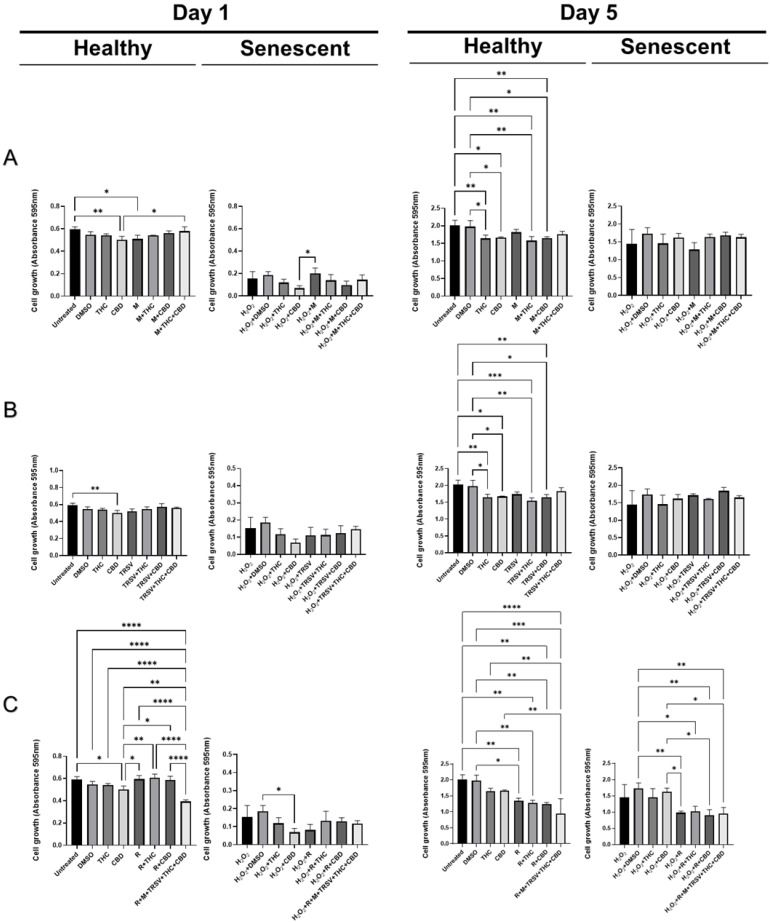
Viability of dermal fibroblasts CCD-1064Sk (p.11) treated with nutrient-signaling regulators combined with phytocannabinoids. The graphs represent cell viability of skin fibroblasts analyzed by MTT assay after experimental treatments with nutrient-signaling regulators combined with THC and CBD. Graphs show cell viability after 1 and 5 days of exposure with (**A**) metformin, (**B**) triacetylresveratrol, and (**C**) rapamycin alone or in combination with phytocannabinoids compared to controls and phytocannabinoids in both the healthy and senescent state. Data were analyzed with a one-way ANOVA test followed by a Tukey post hoc multiple comparison test. Bars represent mean ± SD. Significance is indicated within the figures using the following scale: * *p* < 0.05, ** *p* < 0.01, *** *p* < 0.001, **** *p* < 0.0001. CBD, cannabidiol; DMSO, dimethyl sulfoxide (vehicle), M, metformin; R, rapamycin; THC, Δ-9-tetrahydrocannabinol; TRSV, triacetylresveratrol.

Cell viability of healthy fibroblasts exposed to metformin, TRSV, or rapamycin combined with pCBs for one day was not significantly different from the dimethyl sulfoxide (DMSO) control (Figure 2). At the same time, a mixed treatment of metformin + TRSV + rapamycin + THC + CBD significantly inhibited cellular viability after one day of exposure (Figure 2C). Compared to healthy cells, senescent cells had lower cell viability as expected. In contrast to purported health benefits of CBD, we discovered a significant CBD-induced inhibition of SIPS fibroblasts viability (Figure 2C). Similarly, this trend was repeated but not significant in other experiments (Figure 2A,B). 

Following five days of exposure, NSRs combined with cannabinoids, such as metformin + THC, metformin + CBD, TRSV + THC, TRSV + CBD, rapamycin + THC, rapamycin + CBD, and rapamycin alone decreased cellular viability of healthy cells (Figure 2). Additionally, the viability of senescent dermal fibroblasts was not affected by metformin or TRSV combined with pCBs (Figure 2A,B). In parallel, we identified a significant reduction in cellular growth induced by rapamycin, rapamycin + THC, rapamycin + CBD, and metformin + TRSV + rapamycin + THC + CBD treatments compared to the DMSO control (Figure 2C). 

Analysis showed that rapamycin alone and combined with pCBs, as well as metformin + TRSV + rapamycin + THC + CBD, and CBD alone, had a detrimental effect on cellular viability in healthy and/or senescent CCD-1064Sk dermal fibroblasts (Figure S2). Furthermore, none of the NSRs alone or in combination had beneficial effects on cell viability of senescent cells. This surprising finding led us to test out another cellular viability assay, the neutral red (NR) assay.

The NR assay estimates cellular viability based upon the accumulation of the supravital dye neutral red by endocytosis and internalization into the lysosomes [59]. The viability of dermal fibroblasts was tested after 72 h of exposure to the NSRs with or without pCBs. Increased neutral red uptake of healthy cells would suggest increased cell integrity/viability or increased lysosomal uptake. 

**Figure 3:**
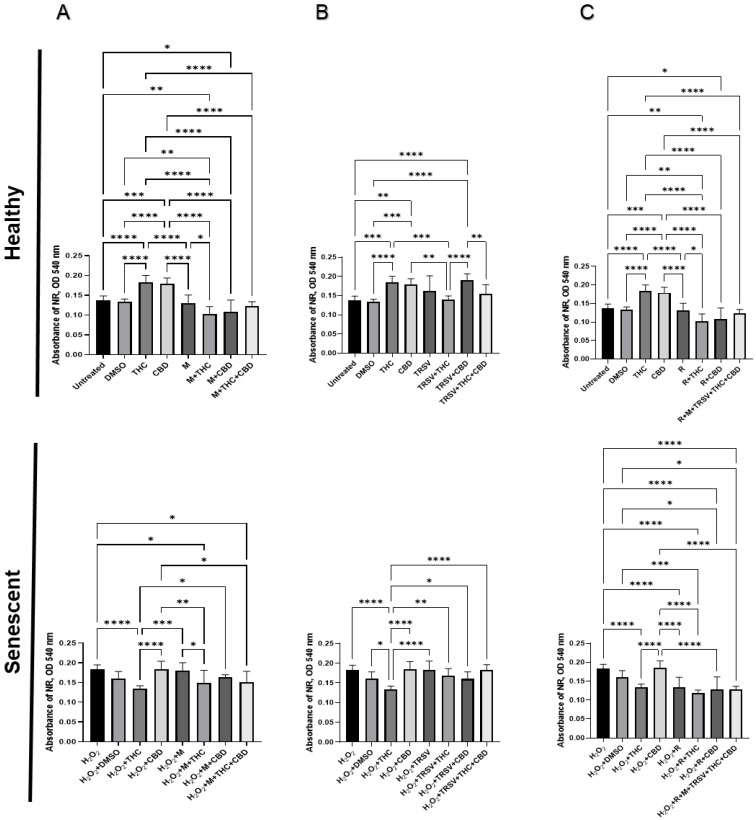
Viability of dermal fibroblasts CCD-1064Sk (p.11) treated with potential anti-aging compounds combined with phytocannabinoids. The graphs represent cell viability of skin fibroblasts estimated by NR assay after 72 h of exposure with (**A**) metformin, (**B**) triacetylresveratrol, and (**C**) rapamycin alone or in combination with phytocannabinoids compared to controls and phytocannabinoids in both the healthy and senescent state. Data were analyzed with a one-way ANOVA test followed by a Tukey post hoc multiple comparison test. Bars represent mean ± SD. Significance is indicated within the figures using the following scale: * *p* < 0.05, ** *p* < 0.01, *** *p* < 0.001, **** *p* < 0.0001. CBD, cannabidiol; DMSO, dimethyl sulfoxide (vehicle), M, metformin; R, rapamycin; THC, Δ-9-tetrahydrocannabinol; TRSV, triacetylresveratrol.

We found increased NR absorbance in healthy cells treated with THC and CBD, and decreased NR absorbance by metformin + THC compared to the DMSO control (Figure 3A). Additionally, TRSV + CBD increased NR absorbance in healthy fibroblasts (Figure 3B), while rapamycin + THC substantially inhibited cell viability (Figure 3C).

SIPS-induced fibroblasts via hydrogen peroxide exposure had consistently higher NR absorbance. This is likely due to increased free radicals from hydrogen peroxide exposure causing damage to the cell membrane and organelles, thereby increasing cell permeability. This would result in increased NR absorbance caused by increased cell permeability. Furthermore, oxidative damage and increased permeability can cause the lysosomes to become activated. In turn, lysosomal overactivation may lead to activation of cell death mechanisms.

In all treatments with metformin and TRSV, NR absorbance in the prematurely aged dermal fibroblasts compared with the DMSO control was not significantly altered (Figure 3A,B). However, rapamycin + THC, rapamycin + CBD, and rapamycin + metformin + TRSV + THC + CBD induced a decrease in NR absorbance compared to the DMSO control (Figure 3C). Due to rapamycin treatments causing significantly reduced cell growth displayed via MTT assay (Figure 2C), this decrease in NR absorbance was likely due to a decrease in cell growth, not due to rapamycin ameliorating the cell membrane/viability. THC exposure without NSRs significantly increased NR absorbance of healthy cells (Figure 3), while it significantly decreased NR absorbance of senescent cells (Figure 3). In contrast, CBD exposure without NSRs significantly increased NR absorbance of healthy cells (Figure 3), while it tended to increase NR absorbance of senescent fibroblasts (*p* > 0.05; Figure 3). As prolonged exposure to pCBs did not affect cell growth of senescent cells (Figure 2), the data would suggest THC may be a better anti-aging therapeutic than NSRs by increasing cell viability of healthy cells and potentially ameliorating cell viability of senescent cells.

### 2.3. The Effect of Phytocannabinoids Combined with Nutrient Signaling Regulators on Wound Healing

Fibroblasts constantly modulate the healing process by repairing and regenerating tissues. Due to the unclear effects of NSRs and pCBs on cell viability, we tested the influence of NSRs and pCBs on the healing functionality of healthy and senescent dermal fibroblasts. For this purpose, scratch lines were created in culture plates of the fibroblasts that represented a wound in the commonly used wound-healing assay (WHA). WHA was done on CCD-1064Sk (p.11) dermal fibroblasts (Figure 4) in the healthy condition and SIPS state. Larger images of each treatment can be seen in the supplementary figures (Figures S3–S18).

The WHA results showed almost complete closure of damaged area in the healthy CCD-1064Sk fibroblasts after 72 h of treatments with or without NSRs and pCBs (Figure 5A). As expected, rapamycin inhibited wound healing (Figure 5B). Co-treatment of rapamycin with THC and CBD significantly improved wound healing (Figure 5B). Metformin alone or in combination with pCBs did not assist wound healing, and metformin alone appeared to inhibit wound healing in healthy fibroblasts (Figure 5B). TRSV appeared to assist wound healing but was not significant (Figure 5B).

**Figure 4:**
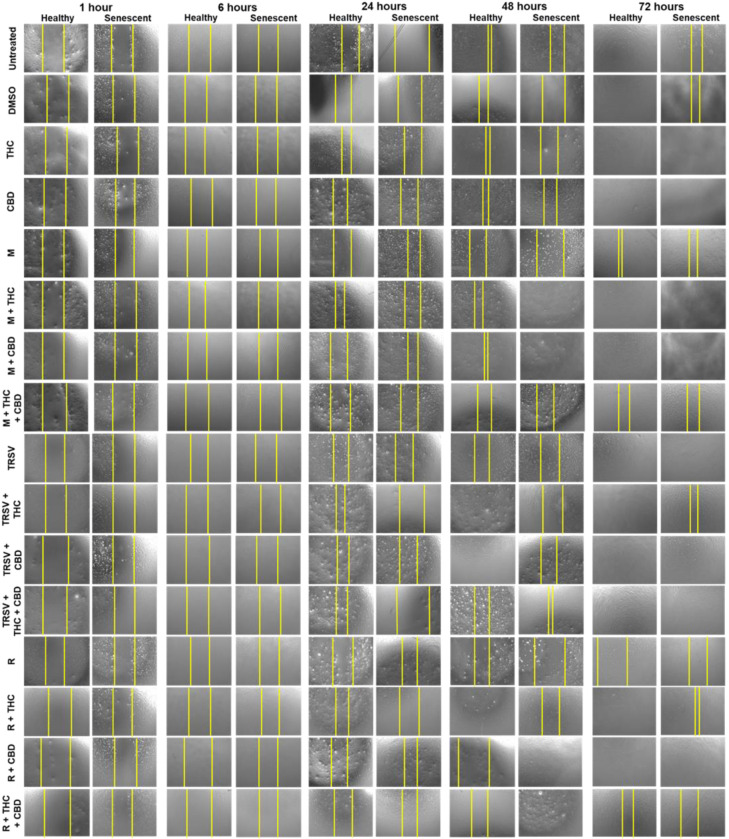
The effect of nutrient signaling regulators combined with phytocannabinoids on human skin fibroblasts CCD-1064Sk (p.11) in the wound-healing assay. Images show size of wounds after exposure to NSRs and/or pCBs for 1, 6, 24, 48, and 72 h in healthy and SIPS fibroblasts. Yellow lines delineate the edge of the wound. CBD, cannabidiol; DMSO, dimethyl sulfoxide (vehicle), M, metformin; R, rapamycin; THC, Δ-9-tetrahydrocannabinol; TRSV, triacetylresveratrol. Images were captured at 80X magnification using a light microscope.

**Figure 5:**
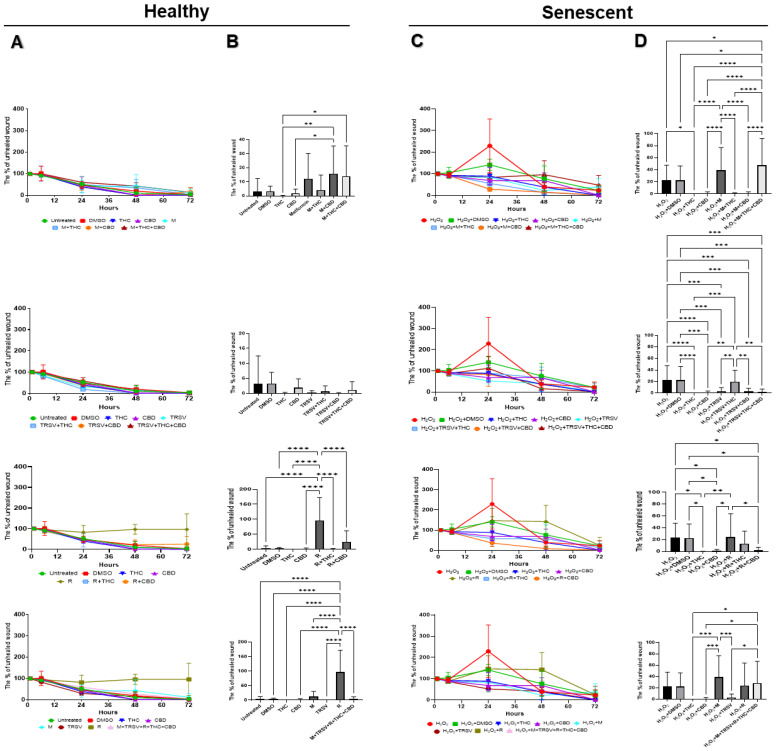
The effect of nutrient signaling regulators combined with phytocannabinoids on wound healing. Graphs represented wound healing assay results in CCD-1064Sk (p.11) dermal fibroblasts. (**A**) the percentage of an unhealed wound in healthy fibroblasts treated with metformin, TRSV and rapamycin combined with pCBs. (**B**) the percentage of the unhealed wound after 72 h of experiment in healthy cells with metformin, TRSV and rapamycin combined with pCBs. (**C**) the percentage of an unhealed wound in senescent fibroblasts culture treated with metformin, TRSV and rapamycin combined with pCBs. (**D**) the percentage of the unhealed wound after 72 h in prematurely aged fibroblasts metformin, TRSV and rapamycin combined with pCBs. Data were analyzed with a one-way ANOVA test followed by a Tukey post hoc multiple comparison test. Bars represent mean ± SD. Significance is indicated within the figures using the following scale: * *p* < 0.05, ** *p* < 0.01, *** *p* < 0.001, **** *p* < 0.0001. CBD, cannabidiol; DMSO, dimethyl sulfoxide (vehicle), M, metformin; R, rapamycin; THC, Δ-9-tetrahydrocannabinol; TRSV, triacetylresveratrol.

As expected, fibroblasts exposed to hydrogen peroxide to induce SIPS had decreased wound healing after 72 h (Figure 4 and Figure 5). Despite reported anti-aging properties of metformin, we found metformin exposure did not improve wound healing, and metformin in combination with THC and CBD significantly inhibited wound healing (Figure 5D). In contrast, metformin in combination with either THC or CBD appeared to improve wound healing compared to the vehicle but was not significant (Figure 5D). TRSV alone, in combination with CBD, or in combination with THC and CBD improved wound healing and appeared to have the most beneficial effects of all tested NSRs (Figure 5A). Surprisingly, rapamycin in combination with CBD increased wound healing speed, but other rapamycin treatments had no change compared to the DMSO control (Figure 5D). Furthermore, exposure to THC or CBD consistently demonstrated significantly increased wound healing speed and appeared to outperform the NSRs.

The knowledge gained from these experiments provides a foundation for investigating the functional roles of NSRs alone in combination with pCBs in dermal healing in an age-dependent manner. The results depicted rapamycin’s adverse effects on the speed of wound healing in senescent cultures. In contrast, TRSV combined with CBD, or metformin combined with THC might be a potential tool for enhancing regeneration and repair in injured tissues. Most surprisingly, THC or CBD alone consistently displayed strong, beneficial results for repair and regeneration in both healthy and senescent injured tissues.

### 2.4. Influence of Nutrient Signaling Regulators and Cannabinoids on the Expression of Cell Cycle Regulators and Metabolic Regulators

Earlier, we reported that senescent cells demonstrate adverse changes in the expression of genes involved in cell-cycle regulation paralleled by changes in cell morphology [54]. Based on our current findings, we decided to test whether metformin, TRSV, rapamycin, and their combinations with pCBs will affect gene expression in healthy and senescent fibroblasts.

The mRNA transcripts levels of cell-cycle progression regulators and senescence-associated markers *CDKN2A* (p16), *CDKN1A* (p21), and *TP53* were measured via quantitative real-time polymerase chain reaction (RT-qPCR) after five days of the experimental treatments. 

**Figure 6:**
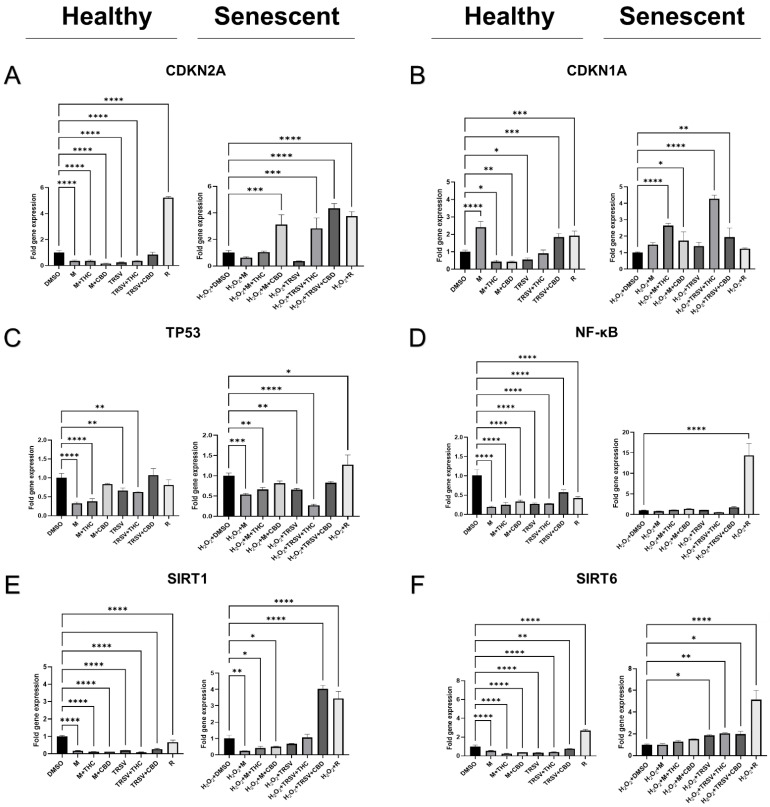
The expression of cellular checkpoint regulators in CCD-1064Sk (p.11) fibroblasts treated with nutrient signaling regulators combined with cannabinoids. Changes of mRNA expression as measured by RT-qPCR for (**A**) *CDKN2A* (p16), (**B**) *CDKN1A* (p21), (**C**) *TP53*, (**D**) *NF-κB*, (**E**) *SIRT1*, and (**F**) *SIRT6* in healthy and senescent fibroblasts. Data were analyzed with a one-way ANOVA test followed by a Dunnett post hoc test compared to the vehicle. Bars represent mean ± SD. Significance is indicated within the figures using the following scale: * *p* < 0.05, ** *p* < 0.01, *** *p* < 0.001, **** *p* < 0.0001. CBD, cannabidiol; DMSO, dimethyl sulfoxide (vehicle), M, metformin; R, rapamycin; THC, Δ-9-tetrahydrocannabinol; TRSV, triacetylresveratrol.

*CDKN2A* levels were significantly upregulated in healthy fibroblasts compared to the vehicle after exposure to rapamycin and decreased following exposure to metformin, metformin + THC, metformin + CBD, TRSV, and TRSV + THC (Figure 6A). In the senescent cells, we observed *CDKN2A* upregulation induced by metformin + CBD, TRSV + THC, TRSV + CBD, and rapamycin (Figure 6A).

In healthy cells, we observed *CDKN1A* upregulation induced by metformin, TRSV + CBD, and rapamycin, whereas metformin + THC, metformin + CBD, and TRSV + THC downregulated *CDKN2A* expression (Figure 6B). In the senescent cells, *CDKN1A* expression increased after exposure to metformin + THC, metformin + CBD, TRSV + THC, and TRSV + CBD (Figure 6B). 

The expression of a critical cellular check-point regulator *TP53* decreased after exposure to metformin, metformin + THC, TRSV, and TRSV + THC in the healthy dermal fibroblasts (Figure 6C). Surprisingly, we discovered rapamycin induced *TP53* upregulation in the senescent cells while metformin, metformin + THC, TRSV, and TRSV + THC significantly decreased *TP53* expression (Figure 6C). 

Next, we looked at one of the prominent regulators of numerous genes associated with cell survival, proliferation, and differentiation expression, *NF-κB*, in healthy dermal fibroblasts in all treatment groups following 5 days of exposure (Figure 6D). Surprisingly, we found increased *NF-κB* mRNA levels in the senescent fibroblasts exposed to rapamycin compared to the vehicle (Figure 6D).

Another essential role of NF-κB is associated with metabolic pathways and is directly related to sirtuin regulation [49]. For this reason, we tested the influence of pCBs combined with NSRs on the mRNA levels of *SIRT1* and *SIRT6* that have been negatively affected in senescent cells in our previous experiments [54]. 

*SIRT1* expression was significantly downregulated in all exposure groups in the healthy fibroblasts (Figure 6E), whereas in senescent fibroblasts, *SIRT1* was downregulated in metformin, metformin + THC, and metformin + CBD treatments while TRSV + CBD, and rapamycin significantly upregulated expression (Figure 6E). In addition, *SIRT6* expression was downregulated in all treatments except rapamycin, which was upregulated in healthy fibroblasts (Figure 6F). *SIRT1*, known as the ‘longevity’ biomarker, was upregulated after exposure to TRSV, TRSV + THC, TRSV + CBD, and rapamycin (Figure 6E). 

Here, we can speculate that rapamycin and TRSV alone or combined with CBD or THC increases expression of pathways associated with cellular growth and metabolic activity in the senescent cells via reduction of cell growth inhibitors p16, p21, and p53, accompanied by potentiation of *NF-κB* expression and corresponding elevation of metabolic biomarkers *SIRT1* and *SIRT6*.

### 2.5. Protective Effects of Nutrient Signaling Regulators and Phytocannabinoids on the Expression of Age-Related Genes Involved in ECM Maintenance

Previously, we have shown SIPS adversely affects ECM components [54], which is in line with the literature. We have shown that functional activity deteriorated and is metabolically reprogrammed in aged dermal cells. These findings encouraged us to test whether NSRs alone or in combination with pCBs have the potential to stimulate collagen and elastin production or preserve it from senescence-associated degradation. Moreover, we also tested whether those treatments changed the expression of cannabinoid receptors.

**Figure 7:**
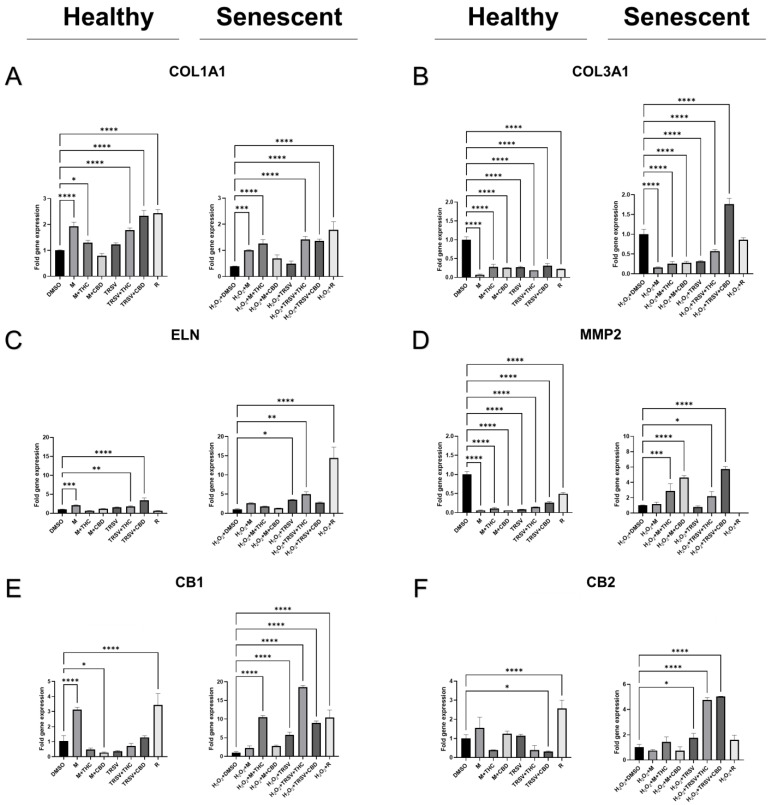
Effects of phytocannabinoids and nutrient signaling regulators on the expression of genes involved in the production of extracellular matrix in CCD-1064Sk (p.11). Changes of mRNA expression as measured by RT-qPCR for (**A**) *COL1A1*; (**B**) *COL3A1*, (**C**) *ELN* (elastin), (**D**) *MMP2*, (**E**) *CB1*, and (**F**) *CB2* in healthy and senescent fibroblasts. Data were analyzed with a one-way ANOVA test followed by a Dunnett post hoc multiple comparison test. Bars represent mean ± SD. Significance is indicated within the figures using the following scale: * *p* < 0.05, ** *p* < 0.01, *** *p* < 0.001, **** *p* < 0.0001. CBD, cannabidiol; DMSO, dimethyl sulfoxide (vehicle), M, metformin; R, rapamycin; THC, Δ-9-tetrahydrocannabinol; TRSV, triacetylresveratrol.

The mRNA level of the dermal collagen type I (*COL1A1*) was increased after exposure to metformin, metformin + THC, TRSV + THC, TRSV + CBD, and rapamycin in healthy fibroblasts, as well as in senescent fibroblasts compared to the DMSO control (Figure 7A). 

Compared to the *COL1A1* data, the *COL3A1* expression had differential expression between the healthy and senescent fibroblasts. The mRNA levels of *COL3A1* in healthy fibroblasts were significantly lowered by all treatments compared to the DMSO control in the healthy fibroblasts (Figure 7B). Meanwhile, in the senescent fibroblasts, metformin, metformin + THC, metformin + CBD, and TRSV + THC significantly lowered *COL3A1* expression compared to the DMSO control. In contrast, TRSV + CBD treatment significantly upregulated *COL3A1* expression compared to the DMSO control (Figure 7B).

In addition to collagens, skin integrity depends on elastin (ELN), which is critical to the elasticity and resilience of many vertebrate tissues, including skin, ligaments, and tendons [60]. In healthy fibroblasts, a substantially increased expression of *ELN* mRNA was noted in response to metformin, TRSV + THC, and TRSV + CBD (Figure 7C). In contrast, TRSV, TRSV + THC and rapamycin significantly upregulated *ELN* expression in the senescent fibroblasts (Figure 7C).

In the microenvironment of healthy cells and tissues, all components of ECM must stay in equilibrium. Most ECM proteins during organogenesis, growth, and normal tissue turnover are degraded by a group of metalloproteinases (MMPs). MMPs are enzymes that are responsible for preventing excessive production and accumulation of healthy or damaged scaffold components. Typically, the expression and activity of MMPs in adult tissues are usually low [61]. In an age-dependent manner, MMP level exponentially increases, and is found in numerous pathological conditions that may lead to unwanted tissue destruction, such as inflammatory conditions, tumor enlargement, and metastasis [62,63]. However, for tissue repair and remodeling of the ECM, increased MMP is required for ECM degradation [64].

We discovered significant inhibition of *MMP2* expression in all treatment groups compared to the DMSO control in healthy fibroblast cells (Figure 7D). However, in senescent fibroblasts, metformin + THC, metformin + CBD, TRSV + THC, and TRSV + CBD upregulated *MMP2* expression (Figure 7D). Given metformin and TRSV in combination with pCBs assists wound healing (Figure 5), the increase in *MMP2* expression is likely a mechanism for repair and regeneration instead of a pathologically overactive MMP2.

Next, we decided to look at cannabinoid receptors (CB)1 and CB2. The canonical cannabinoid receptors are part of the endocannabinoid system (ECS) and belong to the Class A (rhodopsin family) of G-protein coupled receptors (GPCRs) and are known to interact with different phytocannabinoids [65,66]. The expression of these receptors is altered by receptor activation; therefore, the level of expression can serve as a biomarker for a response to cannabinoids [67]. Both *CB1* and *CB2* expression were identified in the normal human skin and its appendages: keratinocytes, hair follicles, sebaceous glands, melanocytes, fibroblasts, nerve fibers, adipocytes, sensory neurons, and immune cells [68]. Therefore, we measured *CB1* and *CB2* mRNA levels to determine if these receptors were likely being altered by NSRs in combination with cannabinoids.

In healthy fibroblasts, we found metformin and rapamycin increased expression of *CB1*, while metformin + CBD significantly inhibited *CB1* expression in healthy dermal fibroblasts (Figure 7E). Similarly, metformin and TRSV appeared to decrease *CB1* expression but was not significant. In contrast, metformin + THC, TRSV, TRSV + THC, TRSV + CBD, and rapamycin significantly augmented *CB1* transcripts levels in prematurely aged fibroblast cells (Figure 7E).

At the same time, mRNA expression of *CB2* in healthy fibroblasts was significantly downregulated after exposure to TRSV + CBD, and significantly upregulated after exposure to rapamycin (Figure 7F). Metformin + THC and rapamycin + THC also showed a trend to decrease *CB2* expression, but it was not significant (Figure 7F). In senescent cells, *CB2* expression increased in TRSV, TRSV + THC, and TRSV + CBD groups compared to the DMSO control (Figure 7F).

Endocannabinoids and their receptors are a constituent part of an adaptive system to regulate cutaneous inflammation; in turn, potentiation of the inflammatory process in the skin is associated with decreased expression of CB1/CB2 [69]. Besides, inflammation is an integral component of the aging process [70]. We can hypothesize that change in the mRNA expression of *CB1* and *CB2* in senescent dermal fibroblasts in response to some NSRs with or without pCBs would affect downstream pathways related to anti-aging mechanisms but needs to be confirmed in future studies utilizing receptor overexpression, knockout, or knockdown.

### 2.6. Effects of Nutrient Signaling Regulators and Phytocannabinoids on the Nuclear Morphology

Based on our earlier study, we discovered nuclear alterations in senescent fibroblasts [54]. Therefore, we decided to test the effect of NSRs alone and combined with pCBs on changes in nuclear morphology utilizing a 4′,6-diamidino-2-phenylindole (DAPI) stain. Previous research showed SIPS caused increased nuclear area, perimeter, min caliper, and max caliper in CCD-1064Sk fibroblast cells [54]. Initially, we compared the nuclear parameters of CCD-1064Sk fibroblast cells exposed to metformin (500 μM), TRSV (10 μM), rapamycin (5 μM), THC (2 μM) and CBD (2 μM). We found significant differences in area, circularity, and perimeter between multiple treatment groups (Figure S19). Specifically, pCBs, metformin, and TRSV appeared to ameliorate changes induced in senescent cells (Figure S19). pCBs alone appeared to have ameliorative effects on all three parameters (Figure S19); however, the focus of our study is specifically on NSRs with or without pCBs. Future studies should focus on the effects of only pCBs on anti-aging properties.

**Figure 8:**
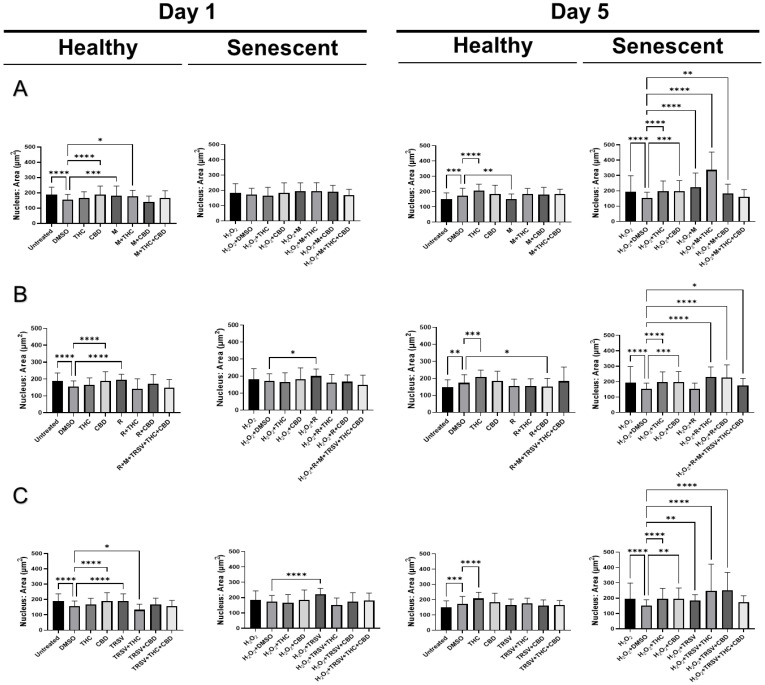
DAPI-stained nuclei area parameters of skin fibroblasts CCD-1064Sk (p.11) exposed to metformin, triacetylresveratrol, and rapamycin combined with pCBs. Nuclear parameters were observed by immunofluorescence microscopy for healthy and senescent fibroblasts treated with (**A**) 500 μM metformin, (**B**) 5 μM rapamycin, or (**C**) 10 μM triacetylresveratrol and combinations with pCBs compared to 2 μM of THC, 2 μM of CBD, DMSO, and untreated fibroblasts after 1 day and 5 days of exposure. Data were analyzed with a one-way ANOVA test followed by a Dunnett’s post hoc test compared to the DMSO or H_2_O_2_ + DMSO control. Bars represent mean ± SD. Significance is indicated within the figures using the following scale: * *p* < 0.05, ** *p* < 0.01, *** *p* < 0.001, **** *p* < 0.0001. CBD, cannabidiol; DMSO, dimethyl sulfoxide (vehicle), M, metformin; R, rapamycin; THC, Δ-9-tetrahydrocannabinol; TRSV, triacetylresveratrol.

**Figure 9:**
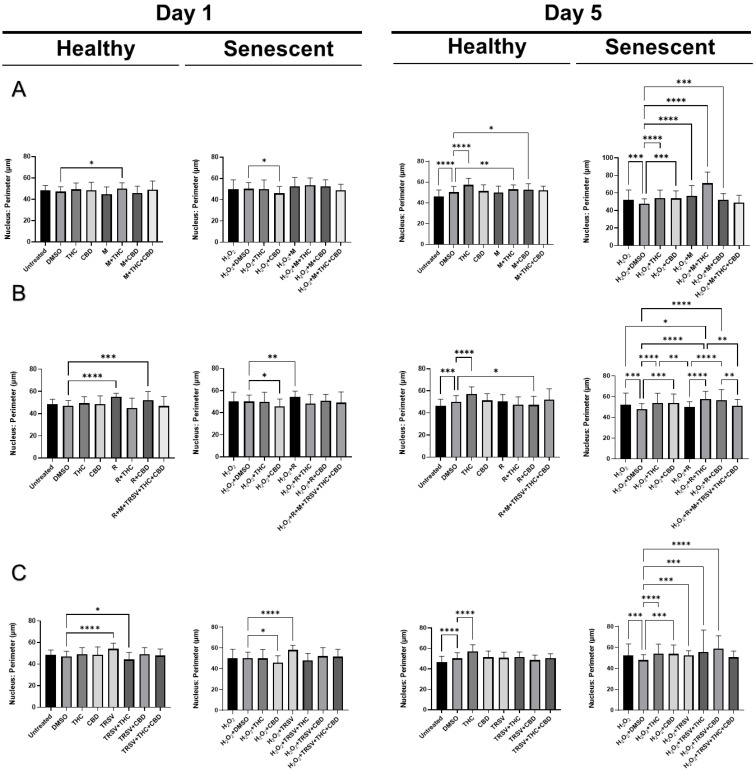
DAPI-stained nuclei perimeter parameters of skin fibroblasts CCD-1064Sk (p.11) exposed to metformin, triacetylresveratrol, and rapamycin combined with pCBs. Nuclear parameters were observed by immunofluorescence microscopy for: healthy and senescent fibroblasts treated with (**A**) 500 μM metformin, (**B**) 5 μM rapamycin, or (**C**) 10 μM triacetylresveratrol and combinations with pCBs compared to 2 μM of THC, 2 μM of CBD, DMSO, and untreated fibroblasts after 1 day and 5 days of exposure. Data were analyzed with a one-way ANOVA test followed by a Dunnett’s post hoc test compared to the DMSO or H_2_O_2_ + DMSO control. Bars represent mean ± SD. Significance is indicated within the figures using the following scale: * *p* < 0.05, ** *p* < 0.01, *** *p* < 0.001, **** *p* < 0.0001. CBD, cannabidiol; DMSO, dimethyl sulfoxide (vehicle), M, metformin; R, rapamycin; THC, Δ-9-tetrahydrocannabinol; TRSV, triacetylresveratrol.

**Figure 10:**
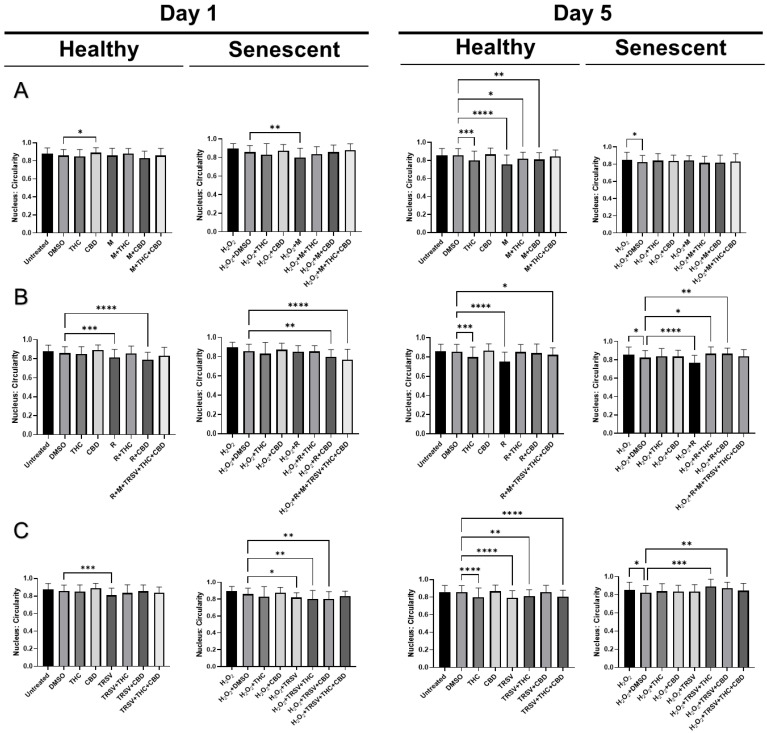
DAPI-stained nuclei circularity parameters of skin fibroblasts CCD-1064Sk (p.11) exposed to metformin, triacetylresveratrol, and rapamycin combined with pCBs. Nuclear parameters were observed by immunofluorescence microscopy for: healthy and senescent fibroblasts treated with (**A**) 500 μM metformin, (**B**) 5 μM rapamycin, or (**C**) 10 μM triacetylresveratrol and combinations with pCBs compared to 2 μM of THC, 2 μM of CBD, DMSO, and untreated fibroblasts after 1 day and 5 days of exposure. Data were analyzed with a one-way ANOVA test followed by a Dunnett’s post hoc test compared to the DMSO or H_2_O_2_ + DMSO control. Bars represent mean ± SD. Significance is indicated within the figures using the following scale: * *p* < 0.05, ** *p* < 0.01, *** *p* < 0.001, **** *p* < 0.0001. CBD, cannabidiol; DMSO, dimethyl sulfoxide (vehicle), M, metformin; R, rapamycin; THC, Δ-9-tetrahydrocannabinol; TRSV, triacetylresveratrol.

**Figure 11:**
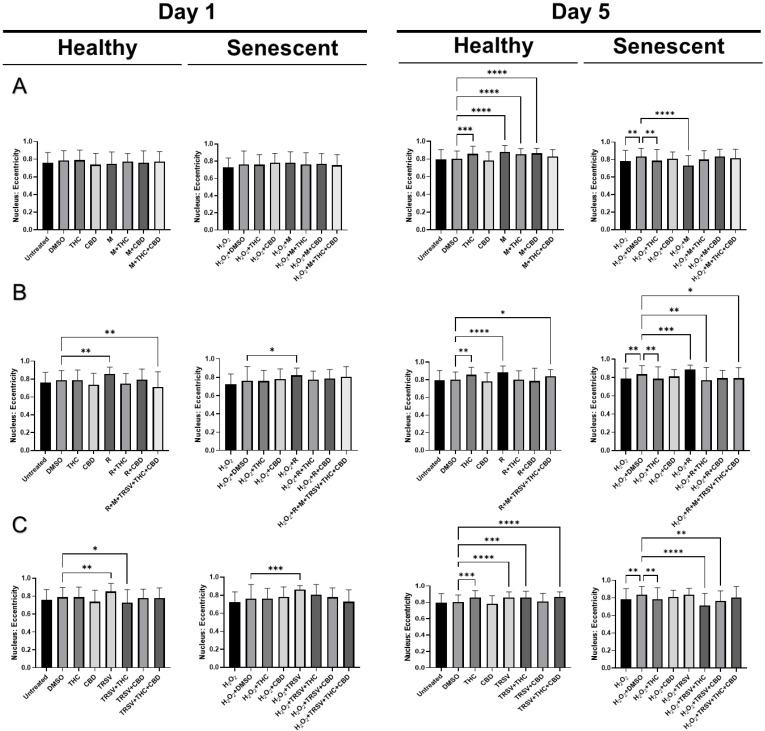
DAPI stained nuclei eccentricity parameters of skin fibroblasts CCD-1064Sk (p.11) exposed to metformin, triacetylresveratrol, and rapamycin combined with pCBs. Nuclear parameters were observed by immunofluorescence microscopy for: healthy and senescent fibroblasts treated with (**A**) 500 μM metformin, (**B**) 5 μM rapamycin, or (**C**) 10 μM triacetylresveratrol and combinations with pCBs compared to 2 μM of THC, 2 μM of CBD, DMSO, and untreated fibroblasts after 1 day and 5 days of exposure. Data were analyzed with a one-way ANOVA test followed by a Dunnett’s post hoc test compared to the DMSO or H_2_O_2_ + DMSO control. Bars represent mean ± SD. Significance is indicated within the figures using the following scale: * *p* < 0.05, ** *p* < 0.01, *** *p* < 0.001, **** *p* < 0.0001. CBD, cannabidiol; DMSO, dimethyl sulfoxide (vehicle), M, metformin; R, rapamycin; THC, Δ-9-tetrahydrocannabinol; TRSV, triacetylresveratrol.

After testing the effect of NSRs and cannabinoids on dermal fibroblasts as separate compounds, we decided to combine NSRs with pCBs to see if pCBs could potentiate the effects of NSRs. Similar to our previous findings [54], nuclei of healthy CCD-1064Sk fibroblasts were round and equal in size while we could qualitatively see an increase in size and change in shape of nuclei in aged fibroblasts (Figures S20–S22). After 1 day of exposure in healthy cells, few alterations in nuclear architecture were seen (Figure 8). Increased nuclear area (Figure 8A) and perimeter (Figure 9A) was seen in metformin and metformin + THC treatments compared to the vehicle, but no changes were seen in circularity (Figure 10A) and eccentricity (Figure 11A). After 5 days of exposure, eccentricity was significantly increased (Figure 10A) and circularity was decreased (Figure 11A) in metformin, metformin + THC, and metformin + CBD treatments compared to the vehicle. Potentially, these alterations help explain the nonsignificant trend of metformin with or without pCBs appearing to inhibit wound healing in healthy fibroblasts (Figure 5B). In contrast, exposure to metformin with or without pCBs for 1 day did not alter the nuclei of senescence fibroblasts (Figure 8A, Figure 10A and Figure 11A), except that metformin exposure reduced circularity (Figure 10B). After 5 days of exposure, metformin with and without pCBs significantly increased area (Figure 8A) and perimeter (Figure 9A), while metformin decreased eccentricity (Figure 11A) in senescent fibroblasts.

Not surprisingly, rapamycin consistently induced changes in nuclear architecture after 1 day of exposure compared to the vehicle, including increased area (Figure 8B), increased perimeter (Figure 9B), decreased circularity (Figure 10B), and increased eccentricity (Figure 11B). These changes in healthy fibroblasts treated with rapamycin are likely indicative of cell damage which is in line with the decreased wound healing ability (Figure 5B) and cell cytotoxicity (Figure 2C). Similarly, rapamycin exposure for 5 days in healthy cells induced significantly decreased circularity (Figure 10B) and increased eccentricity (Figure 11B), demonstrating the nuclei were more oval shaped with altered area-to-perimeter ratios. In senescent fibroblasts, 1 day of exposure to rapamycin similarly increased area (Figure 8B), perimeter (Figure 9B), and eccentricity (Figure 11B), while after 5 days of exposure, rapamycin with pCBs showed similar changes in area (Figure 8B) and perimeter (Figure 9B), while increasing circularity (Figure 10B) and decreasing eccentricity (Figure 11B). Based upon these results, the adverse effects of rapamycin were at least partially mitigated by the addition of phytocannabinoids, which would be in line with our functional data showing rapamycin in combination with either THC or CBD increases wound healing speed compared to rapamycin (Figure 5D). 

TRSV + THC exposure for 1 day in healthy fibroblasts consistently induced nuclear architecture alterations with decreased area (Figure 8C), perimeter (Figure 9C), and eccentricity (Figure 11C). Although no changes were seen in area (Figure 8C) or perimeter (Figure 9C) after 5 days of exposure in healthy fibroblasts, circularity (Figure 10C) and eccentricity (Figure 11C) were significantly lower in the TRSV and TRSV + THC groups compared to the vehicle. This result was surprising as no noticeable differences in healthy fibroblast wound healing were seen between treatments (Figure 5B). In contrast, nuclear architecture was significantly altered in senescent fibroblasts when exposed to TRSV treatment for 1 day but not when combined with pCBs (Figure 8C, Figure 10C, and Figure 11C), except for circularity where all TRSV treatments were decreased, as was observed compared to the control (Figure 10C). After 5 days of exposure, TRSV, TRSV + THC, and TRSV + CBD treatments increased area (Figure 8C) and perimeter (Figure 9C), while TRSV + THC and TRSV + CBD also increased circularity (Figure 10C) and decreased eccentricity (Figure 11C). While TRSV with or without pCBs had the best wound healing out of all NSRs (Figure 5B,D), the increased area and perimeter may be compensatory mechanisms, while decreased eccentricity and increased circularity suggests amelioration of changes in the nuclear architecture by reducing the previously shown hydrogen peroxide-induced elongated phenotype [54]. 

## 3. Discussion

Despite immense research being performed, countless questions remain regarding the role of fibroblasts in dermal aging. Previously, we have shown fibroblasts demonstrate aging by displaying morphological alterations, elevations of biomolecular markers of senescence, changes of gene expression, changes in functionality, and metabolic reprogramming [54]. Our aims were to target the senescence-associated changes in dermal fibroblasts for the development of potential anti-aging and rejuvenation tools.

The current study established the effects of NSRs (metformin, TRSV, and rapamycin) alone and combined with pCBs on the healthy and senescent skin fibroblasts. Based on the cellular viability test, we determined the concentrations of 500 µM for metformin, 10 µM for TRSV, and 5 µM for rapamycin were the optimal doses for anti-aging. The efficacy of NSRs alone and combined with pCBs were tested by cellular viability MTT and NR assays, wound healing assay, RT-qPCR, and nuclear DAPI staining. The results demonstrated that NSRs differentially affected the senescence-associated phenotypes in dermal fibroblasts.

During routine microscopical examinations of prematurely aged fibroblasts, we revealed fibroblast enlargement with evident loss of their elongated spindle-like features, with fibroblasts turning from bipolar into amorphous-like shape [54]. These findings were consistent with the data from other cultures of senescent fibroblasts [71,72]. 

Although numerous studies showed a significant positive influence of metformin, resveratrol, and rapamycin on the viability of senescent cells [46,50,73,74,75,76,77,78], our results have not revealed significant improvements in cellular viability induced by NSRs in combinations with or without pCBs (Figure 2 and Figure 3). Furthermore, we discovered that rapamycin alone and combined with THC or CBD, and a mixed treatment that encompassed metformin, TRSV, rapamycin, THC, and CBD exerted a detrimental effect in both cellular viability assays in healthy and senescent dermal fibroblasts (Figure 2 and Figure 3). This is in contrast to published studies that demonstrated topical rapamycin application reduced senescence and age-related features in human skin [79].

Zhao et al. (2017) also noted that metformin and, to a lesser extent, resveratrol improved the healing process through stimulation of the AMPK pathway, which has been documented to inhibit wound healing [80], probably because of its immunosuppressive capability upon systemic administration [50,76,80]. Our results were generally in line with these findings, suggesting TRSV and metformin could be beneficial for therapeutics in dermal healing.

Wound-healing assays revealed that typically wound closure completed within 72 h (Figure 4 and Figure 5). In healthy fibroblasts, we detected rapamycin delayed wound healing in CCD-1064Sk fibroblasts. These results suggest rapamycin might be considered in treating hyperproliferative disorders like psoriasis and cosmetic manipulations, but not for wound healing or anti-aging therapeutics.

Remarkably, in the senescent CCD-1064Sk fibroblast cultures, we discovered a common trend to potentiate wound healing with the addition of THC or CBD to NSRs. Metformin with THC or CBD produced significantly improved wound healing compared to just metformin, while rapamycin + THC had significantly improved wound healing compared to just rapamycin (Figure 5). TRSV had the best wound healing out of any NSRs in either healthy or senescent fibroblasts (Figure 5B,D), while also not appearing to be improved by the addition of pCBs (Figure 5B,D). In fact, TRSV + THC had decreased wound healing ability compared to TRSV alone in senescent fibroblasts (Figure 5D). However, metformin + THC + CBD substantially delayed wound closure compared to the vehicle (Figure 5D). 

Most anti-aging nuclei studies were focused on genome organization and nuclear structure in young, proliferating, and often transformed cells. However, for the analysis of functional activity in replicative and stress-induced senescence, it would be more relevant to analyze the nucleus of non-proliferating quiescent or senescent cells [81]. Our previous results determined significant nuclear alterations in SIPS fibroblasts compared to healthy fibroblasts [54], while, in this study, we showed NSRs with or without pCBs altered nuclear architecture as well (Figure 8, Figure 9, Figure 10 and Figure 11). For instance, the nuclear area, a parameter that allows identifying size changes, was 149.5 µm^2^ in healthy young cells, while it was 194.2 µm^2^ in the SIPS CCD-1064Sk fibroblasts (Figure 8). Other studies reported similar alterations on nuclear architecture of different senescent fibroblast cell cultures. For example, Bridger and Kill (2004) studied nuclear abnormalities in normal dermal fibroblast cultures 1BR and NB1 and compared them with prematurely senescent ones from Hutchinson–Gilford progeria syndrome fibroblasts [82]. They revealed that cellular aging is characterized by a shift in the nuclear area towards larger nuclei. Moreover, it was noted that naturally aged nuclei exerted the same features as Hutchinson–Gilford progeria syndrome fibroblasts [82]. In line with these observations, we also found that senescent fibroblasts have larger nuclei.

We found metformin with and without pCBs altered nuclear architecture in healthy fibroblast cells. Changes included increased area (Figure 8A) and perimeter (Figure 9A) after 1 day of exposure, and increased circularity (Figure 10A) and decreased eccentricity (Figure 11A) after 5 days of exposure. These changes can help explain the nonsignificant trend of inhibited wound healing in healthy fibroblasts (Figure 5B). In contrast, metformin with or without pCBs generally did not affect nuclear architecture after 1 day of exposure. After 5 days of exposure, metformin with and without pCBs significantly increased area (Figure 8A) and perimeter (Figure 9A), while metformin decreased eccentricity (Figure 11A) in senescent fibroblasts. Since metformin with pCBs had significantly better wound healing than metformin alone (Figure 5D), it is difficult to understand why no major nuclear changes were seen between these groups unless nuclear architecture is not an important factor in the functional activity of metformin with pCBs. 

A recent study reported dose-dependent resveratrol-induced premature senescence in dermal fibroblasts, based on increased β-Gal staining, increased expression of p16, p21, and p53 cell-cycle inhibitors, and accumulated senescence-associated heterochromatic foci (SAHF), known as areas of condensed and transcriptionally silenced DNA [83]. They found that the decline in proliferative activity was associated with resveratrol doses higher than 10 μM and that resveratrol induced apoptosis at 100 μM. The percentage of apoptotic cells was increased to 8.3 ± 1.5 at 100 μM and reached to 37 ± 1.5 after usage of 200 μM and 300 μM of resveratrol, while directly correlating with elevated SAHF appearance. These findings are essential considering the safety and efficacy of resveratrol. In contrast, our studies used 10 μM TRSV, an acetylated form of resveratrol with reportedly higher bioavailability [46,47]. 

Differences in nuclear architecture were apparent between TRSV and TRSV with pCBs after 1 and 5 days of exposure in healthy fibroblasts; however, no noticeable differences in healthy fibroblast wound healing were seen between treatments (Figure 5B). In contrast, nuclear architecture was significantly altered in senescent fibroblasts for TRSV, TRSV + THC and TRSV + CBD treatments after 5 days of exposure. Since TRSV with or without pCBs had the best wound healing out of all NSRs (Figure 5B,D), the data suggest the increased area and perimeter induced by TRSV, TRSV + THC, and TRSV + CBD in senescent fibroblasts may be compensatory mechanisms, while decreased eccentricity and increased circularity suggests amelioration of the nuclear architecture by reducing elongated phenotype that was previously shown to be induced by hydrogen peroxide [54]. Thus, we can speculate that TRSV possesses geroprotective activity toward senescent nuclei by ameliorating nuclear architecture.

Last, rapamycin exposure alone generally altered nuclear architecture, while the addition of pCBs generally returned nuclear architecture parameters to those observed in the vehicle (Figure 9, Figure 10 and Figure 11). These changes induced by rapamycin are detrimental as shown by the corresponding wound healing data (Figure 5B,D), while addition of THC or CBD appear to ameliorate these changes induced by rapamycin (Figure 8 and Figure 10 and Figure 11) and further support the use of THC or CBD in addition to NSRs. 

The structural components of the nucleus, nuclear envelope, nucleoli, nuclear bodies, and the nuclear matrix, are intimately linked to the genome, maintaining signaling and ultimately control of the genome functionality [82]. Correspondingly, disorganization or defects in nuclear structures and/or architecture are associated with alterations of normal genome regulation and are commonly found in cancer and severe premature aging diseases, like progeroid syndromes and laminopathies [84]. This is also supported by the observation of changes in the expression of cell-cycle regulators involved in potentiating senescence phenotypes such as p16, p21, and p53. They are engaged in two well-known theoretical signaling transduction mechanisms responsible for cellular senescence, the p53-p21-pRb pathway and the p16-pRb pathway [85]. 

NSRs and pCBs significantly affected both senescence pathways: the telomere-based p53-p21-pRb, which typically represents replicative senescence (RS), and the stress-based p16-pRB pathway, which represents SIPS [86]. The inhibitory effects of TRSV and its combinations with pCBs on *TP53*, *CDKN2A* (p16), and *CDKN1A* (p21) discovered in our studies are in line with recent studies on the anti-aging properties of resveratrol [85,87]. For example, adding 30 µM and 60 µM of resveratrol partly downregulated the expression of two senescence markers (p16 and p53) in a concentration-dependent manner, suggesting that resveratrol can suppress mechanical overloading-induced nucleus pulposus cell senescence [85]. Furthermore, 4 weeks application of 5 µM of resveratrol on an in vitro senescence model of human MRC5 lung fibroblasts lowered the levels of p53 acetylation by about 35% in RS cells (PDL > 50) [87]. Our data show TRSV similarly downregulates *CDKN1A*, *CDKN2A*, and *TP53* (Figure 6). The addition of pCBs generally prevented these changes or upregulated mRNA expression (Figure 6). Whether or not these changes are reflected in the encoded proteins remains to be investigated.

Furthermore, Zhao et al. (2017) showed metformin-induced mRNA upregulation of cyclin D1 (CCND1), a proliferative promoter, and downregulated cell cycle inhibitors P53, P21, and P16, in wounds of aged mice [50]. In parallel, they noted resveratrol was less potent in CCND1 promotion and inhibition of P53, P21, and P16 than metformin. Moreover, the authors admitted paradoxical suppressive effects of rapamycin on P53 and P21 but stimulatory effects on P16, which might cause the increased CCND1 levels. Both metformin and resveratrol demonstrated an increase in the viability of proliferative cells alongside the epidermis and around hair follicles, ameliorated aging in cutaneous wound healing, with metformin having a more profound anti-aging effect [50]. Our data on metformin and pCBs’ effect on cell cycle regulators is not uniform with metformin, generally decreasing *CDKN2A*, increasing *CDKN1A*, and decreasing *TP53* mRNA expression. 

Based on the free-radical theory of aging, oxidative stress caused by ROS accumulation can potentiate cellular senescence [54]. In the present study, we also analyzed the effects of NSRs and pCBs on NF-κB activation induced by ROS in dermal fibroblasts exposed to hydrogen peroxide to induce senescence [88]. 

In healthy dermal fibroblasts, *NF-κB* was downregulated in all treatment groups following 5 days of exposure (Figure 6D). We also detected increased mRNA levels of *NF-κB* in the rapamycin treatment in SIPS fibroblasts compared to the vehicle (Figure 6D). However, *NF-κB* transcripts in senescent fibroblasts were lower than the corresponding levels in healthy cells. There is a substantial body of evidence showing the transcriptional activity of NF-κB is increased in aged tissues and associated with multiple age-related degenerative diseases, including Alzheimer’s, diabetes, and osteoporosis [40]. Furthermore, Tilstra and colleagues reported that NF-κB inhibition leads to delayed onset of age-related symptoms and pathologies in murine models [89]. They also pointed out that stimulation of NF-κB is involved in the supervision of numerous known lifespan regulators, including insulin/IGF-1, FOXO, SIRT, mTOR, and DNA damage. Thus, the inhibitory effect of all treatments on healthy cells might provide anti-aging effects of healthy skin (Figure 6D); however, in senescent cells, no treatments significantly reduced *NF-κB* expression. Furthermore, *NF-κB* expression was strongly upregulated in the rapamycin exposure of senescent cells. This could be one mechanism that prevents rapamycin from providing any potential beneficial anti-aging effects in skin (Figure 5D).

There is a large body of data supporting the involvement of NF-κB in regulating metabolic pathways directly linked to sirtuin modulation [34,49,50,89]. The leading role of sirtuins on the protection from cellular senescence has mainly been intimately associated with SIRT1 and SIRT6 [90]. SIRT1 is predominately found in the nucleus and shuttles between the cytoplasm and nucleus and is highly involved in cellular senescence and aging via enzymatic activity of deacetylase resulting in adenosine-diphosphate-ribosyl-transferase triggered DNA repair [6,38,91,92]. SIRT6 is primarily localized in the heterochromatic regions and modulates lifespan extension via adenosine-diphosphate-ribosylation activities, while its deacetylase activity is focused on DNA repair, genome stability, and telomere maintenance [92].

Sirtuins modulate the organismal lifespan by interacting with several lifespan regulating signaling pathways, including the insulin/IGF-1 signaling pathway, TOR, AMPK, FOXO, and the NAD+-dependent sirtuin deacetylases that are commonly dysregulated in aging and ARDs [90,93,94]. Furthermore, the promotion of premature senescence-like phenotypes in endothelial cells was induced by the reduction of SIRT1 and SIRT6 through siRNA or miRNA targeting via pharmacological inhibitors [2]. On the other hand, overexpression of SIRT1 [95,96] or inhibition of SIRT6 [97,98] reduces senescence biomarkers in multiple different cell models.

Our previous studies identified the dysregulation of multiple SIRT family members in SIPS dermal fibroblasts [54]. During this study, we found *SIRT1* was downregulated by metformin, metformin + THC, and metformin + CBD but upregulated by TRSV + CBD after 5 days of exposure in senescent fibroblasts (Figure 6). In addition, *SIRT6* was upregulated for TRSV, TRSV + THC, TRSV + CBD, and rapamycin after 5 days of exposure. These results are somewhat contradictory as SIRT1, the ‘longevity’ biomarker, mRNA expression would suggest TRSV + CBD are best for anti-aging treatments; however, increased *SIRT6* expression would suggest otherwise. In contrast, in healthy cells, *SIRT1* was decreased in all treatments, while *SIRT6* was decreased in all treatments except rapamycin, which upregulated *SIRT6* (Figure 6). Further work should be performed on protein expression to confirm the results demonstrated here.

Resveratrol was reported to exert neuroprotective effects by modulation of mitochondrial function, and the elimination of mutant proteins associated with neurodegenerative diseases by enhancement of autophagic and lysosomal clearance of Aβ-amyloid. These effects were found to occur by induction of SIRT1 and AMPK activation leading to mTOR suppression [9,74,86]. Moreover, NSRs were found to reduce the concentrations of the hormonal effectors such as insulin, IGF1, and growth hormone, which stimulated or inhibited the activity of numerous metabolic sensors (i.e., sirtuins, AMPK, TOR). Bonkowski and Sinclair (2016) showed that sirtuin-activating compounds such as SRT1720 and SRT2104 can directly activate SIRT1, whereas rapamycin is a direct inhibitor of mTOR, and metformin indirectly activates AMPK. In addition, these metabolic regulators mediate downstream activities such as DNA repair, mitochondrial biogenesis and function, stress resistance, stem cell and telomere maintenance, autophagy, chromatin modifications, reduced inflammation, and translation fidelity [93]. In addition, the inhibitory effects of resveratrol on the mTOR regulation, accompanied by potentiation of autophagic and lysosomal activities in senescent cells, might explain our findings related to increased lysosomal NR accumulation and enhanced cellular viability in prematurely senescent fibroblasts exposed to TRSV and pCBs (Figure 3). Altogether, these activities improved cellular homeostasis, reduced morbidity, and presented a more youthful-like state [77,93].

These results supported the idea that NSRs in combination with pCBs exerted protective activity in cellular senescence and might be used as anti-aging remedies by improving the metabolic functionality in prematurely aged dermal fibroblasts. Specifically, our data suggest TRSV or metformin would likely provide more beneficial effects than rapamycin.

Apart from the metabolic regulation, the AMPK pathway also modulates fibroblasts’ functional activity linked to the generation of collagen and other ECM components [50]. The UV-induced elevation of MMP1 expression in human dermal fibroblasts was related to the potentiation of mTOR phosphorylation. In contrast, augmented phosphorylation of AMPK and liver kinase B1, which are mTOR inhibitors, led to suppression of MMP1-initiated activity towards ECM degradation [99]. Metformin was reported to ameliorate age-related thinning of the epidermis, reduction in hair follicle number, decreased collagen deposition, and downregulation of p-acetyl-CoA carboxylase and p-AMPK in the epidermis [50]. 

Notably, our results discovered effects of NSRs combined with pCBs on gene expression in healthy fibroblasts (Figure 7). A prominent increase in *COL1A1* expression was induced by metformin, metformin + THC, TRSV + THC, TRSV + CBD, and rapamycin in both healthy and senescent cells (Figure 7A). Unfortunately, significantly decreased *COL3A1* expression was observed in all treatments on healthy fibroblasts, and most treatments also decreased mRNA expression in senescent fibroblasts (Figure 7B). TRSV + CBD increased *COL3A1* and *COL1A1* expression in both the senescent and healthy fibroblasts, suggesting TRSV + CBD may be the best treatment option for collagen production. Once again, our data would suggest NSRs in combination with pCBs provide more therapeutic value than NSRs alone.

These changes are likely to be related to ROS-induced excessive protein glycation causing the generation of AGEs, which contribute to skin aging as it deteriorates the existing collagen by crosslinking. Besides, the progressive increase of AGEs during aging causes oxidative damage to cellular macromolecules and modulates the activation of transcription factor NF-κB resulting in apoptosis enhancement and ceased proliferative activity [15]. This assertion aligns with our experimental findings, where *NF-κB* was predominantly upregulated in SIPS fibroblasts and downregulated in multiple treatment groups (Figure 6D).

Another crucial part of the dermal extracellular matrix is ELN. Elastin helps to retain elasticity and regain ECM shape after stretching or contracting. In healthy fibroblasts, mRNA levels of *ELN* were upregulated by metformin + THC, TRVS + THC, and TRSV + CBD exposure, while TRVS, TRSV + THC also upregulated *ELN* in senescent cells (Figure 7C). For *ELN* mRNA expression, our results demonstrated TRSV alone or in combination with pCBs produced beneficial effects, especially TRSV + THC. 

Meanwhile, matrix metalloproteinases degrade collagen, elastin, and other components of the extracellular matrix. While overactive MMPs can be detrimental, it is important to note MMPs have a physiological role for healthy ECM remodeling in tissue repair and wound healing. We noticed decreased *MMP2* in healthy cells after exposure to all treatments (Figure 7D). As healthy cells do not need high levels of MMPs to remodel the ECM, this shows all treatments had beneficial effects. In senescent cells, we noticed metformin + THC, metformin + CBD, TRSV + THC, and TRSV + CBD upregulated *MMP2* levels (Figure 7D). Upregulation of *MMP2* levels is likely beneficial and help support ECM remodeling especially considering that most treatments greatly upregulated mRNA expression of *COL1A1* and *COL3A1* as well.

In recent studies, metformin is often mentioned as a collagen protector in aging and high glucose-associated conditions. For instance, 24 h of treatment with 500 μM of metformin significantly increased collagen I and III generation in aged 3T3 fibroblasts, while the activity of MMPs was decreased [15,16]. Similar results were reported by Shao et al. (2014)—metformin had a direct effect on osteoblast-like cells MG63 and attenuated the suppression of proliferation induced by high glucose with increased expression of collagen type I, osteocalcin, osteoprotegerin, while suppressing MMP1 and MMP2 [75]. In contrast, Benazzoug et al. (1998) have reported that glucose specifically increases collagen type III synthesis both at the mRNA and protein levels, without alteration of collagen type I production in young and replicative senescent cultured human skin fibroblasts [73]. Furthermore, ceramides have been found to prevent ECM atrophy and increase dermal functionality via ECM remodeling through upregulation of elastin and MMP2 through a TGF-β mechanism [100]. In conjunction with our findings, positive effects of TRSV might be mediated via the SIRT1 pathway component—peroxisome proliferator-activated receptor γ coactivator, accompanied by NAD+ elevation and AMPK pathway stimulation [78]. 

Senescence is inseparably accompanied by the inflammatory process, ROS-induced gradual damage of cellular components and deterioration of molecular signaling pathways, and alteration of cell-cycle and metabolic regulation that eventually leads to increased growth and cell death. Nevertheless, stimulation or inhibition of specific components of ECS, the influence of endocannabinoids (eCBs), or pCBs have the potential to delay or alleviate this process targeting multiple components of the vicious cycle of aging. The main targets of eCBs/pCBs are cannabinoid receptors CB1, which is associated with the analgesic effects of cannabinoids, and CB2, which modulates cytokine release from immune cells and reduces inflammation [101]. In the skin, cannabinoid receptor antagonists exacerbate allergic inflammation, whereas receptor agonists attenuate inflammation. Pharmacological inhibition or knockout of CB1/CB2 receptors in mice augmented the hapten dinitrofluorobenzene-induced dermatitis and was linked to the increased levels of eCBs, anandamide, and 2-arachidonoylglycerol. In addition, dinitrofluorobenzene treatment decreases *CB1* mRNA and increases *CB2* mRNA [102]. Moreover, reduced expression of cannabinoid receptors was observed in contact dermatitis [69]. At the same time, CB1 activation suppressed the expression of two damage-induced keratins, keratin 6 and keratin 16, which are highly upregulated in hyperproliferative disorder psoriasis [103]. Therefore, we measured mRNA levels of *CB1*/*CB2*. In healthy dermal fibroblasts, we identified metformin and rapamycin stimulated *CB1* expression, while metformin + THC inhibited *CB1* (Figure 7E). In contrast, metformin + THC, TRSV, TRSV + THC, TRSV + CBD, and rapamycin upregulated *CB1* expression (Figure 7E). Furthermore, TRSV + CBD decreased *CB2* expression in healthy fibroblasts while TRSV, TRSV + THC, and TRSV + CBD increased *CB2* expression in senescent fibroblasts (Figure 7F). Alteration of expression of *CB1* and *CB2* would suggest altered activity of these receptors. As expression was increased in both receptors by TRSV, TRSV + THC, and TRSV + CBD in senescent cells, there would likely be higher receptor activity and attenuated inflammation as a result. 

We believe TRSV in combination with CBD provides the best potential anti-aging therapeutic because of increased cell viability, restored wound healing functional activity, reduced metabolic dysfunction, and ameliorated nuclear parameters. Although the mechanism of how TRSV + CBD achieves this has not been elucidated, this likely occurs through the CB1 receptor. Our data shows all TRSV groups significantly altered *CB1* and *CB2* mRNA expression in senescent fibroblasts, but not in healthy fibroblasts. Resveratrol has affinity for CB1 acting as a neutral antagonist [104]. It is possible that overactive CB1 signaling in the senescent state could be ameliorated with either TRSV alone or TRSV + CBD, but not TRSV + THC. Indeed, we show that TRSV + THC significantly inhibits wound healing compared to either TRSV alone or TRSV + CBD and is comparable to the vehicle. Although beneficial effects of CBD may be established through the CB2 receptor, it is unlikely TRSV’s beneficial effects occur through the CB2 because of resveratrol’s fivefold selectivity for CB1 over CB2 [104]; however, this cannot be excluded. Furthermore, TRSV, which has a bioavailability of 80% compared to 29.7% for resveratrol, may also be beneficial, as metabolites of resveratrol/TRSV appear to have no affinity for CB1 [104]. Further research to validate the mechanism of action should be performed. 

Since many polyphenols derived from natural sources have low solubility, low bioavailability, and poor stability [105,106], there has been a growing trend to utilize nano-formulations and emulsions to bypass the limiting pharmacokinetics of each polyphenol and increase efficacy [107,108,109]. Although TRSV has 80% bioavailability when taken orally, these technologies could still help decrease metabolism and increase the half-life of TRSV, which is only 104 min [110]. Rapamycin could similarly benefit from nano-formulations and emulsions to prevent or minimize first-pass metabolism because of the limited bioavailability of 14% despite a long half-life of 68 h [111]. In addition, metformin is not metabolized by the liver and has a bioavailability of ~50% with a half-life between 4 and 8.7 h [112]. Furthermore, TRSV and CBD could be combined with nano-formulations or/and emulsions to produce one pill that simplifies administration. Future studies should test the efficacy of emulsions and nano-formulations of TRSV and CBD in vivo.

## 4. Materials and Methods

### 4.1. Cell Culture and Maintenance

Healthy human neonatal foreskin fibroblasts CCD-1064Sk (ATCC^®^ CRL-2076™), were obtained from the American Type Culture Collection (Rockville, MD, USA). Cells were cultivated in ISCOVE’s Modified Dulbecco’s Medium (IMDM) 1X (MULTICELL, Cat# 319-106-CL) containing 10% heat-inactivated premium grade fetal bovine serum (Cat# 97068-085, VWR International LLC, Radnor, PA, USA), and 1% penicillin-streptomycin (10,000 IU penicillin and 10,000 ug/mL streptomycin, Cat# 450-201-EL, Wisent Inc., Saint-Jean-Baptiste, QC, Canada). All cells were grown and harvested in our BSL-2 laboratory at the University of Lethbridge. Experimental cell lines were incubated in a humidified Forma Steri-Cycle CO_2_ Incubator (Thermo Fisher Scientific, Waltham, MA, USA) at 37 °C with 5% CO_2_. Cell culture media were replaced with fresh media every three days until cell confluency reached 90–100% for further experiments. The cells were subcultured every six or seven days. The replication speed or population doubling (PD) numbers of the cell lines were determined for each subculture as ΔPD = log2(n_f_/n_i_), where n_i_ is the number of cells initially seeded and n_f_ is the final number of cells in a culture. Cells for the senescence model were not older than 24–30 population doublings when employed in the experiments.

### 4.2. Senescence-Associated Phenotype Modeling

Skin fibroblasts (CCD-1064Sk) at 70% confluency were treated for 1 h with 25 µM concentration of hydrogen peroxide solution (H_2_O_2_ dissolved in D-PBS) in 100 × 15 mm petri plates in aseptic conditions (Figure 1). Petri plates with skin fibroblasts were maintained in a humidified incubator at 37 °C with 5% CO_2_.

After a single 1 h treatment, H_2_O_2_ solution was poured out and substituted with cell culture medium. Subsequently, SIPS features and biomarkers were determined via microscopy, MTT, western immunoblotting, reverse transcription-polymerase chain reaction, wound-healing assay, and nuclear DAPI staining.

### 4.3. Experimental Anti-Aging Treatments 

Fibroblast cell cultures were treated with cannabinoids THC and CBD at 2.0 µM concentration. In addition, CCD-1064Sk was treated with three popular anti-aging drugs: rapamycin (Thermo Fisher Scientific, Waltham, MA, USA), metformin (Cedarlane, Toronto, ON, Canada,), and TRSV (VWR, Radnor, PA, USA). The concentrations of the anti-aging compounds were based on an accumulated body of literature that has demonstrated efficacy in anti-aging studies, for rapamycin 1 µM, 5 µM, 10 µM, 50 µM [19], for metformin 50 µM, 100 µM, 500 µM [15], and for TRSV 5 µM, 10 µM, 50 µM [35,44,46]. All anti-aging drugs were dissolved in DMSO due to high solubility: up to 25 mg/mL for TRSV, up to 200 mg/mL for rapamycin, and greater than 300 mg/mL for metformin. Next, rapamycin, metformin, TRSV, CBD, THC, and a vehicle (DMSO) were dissolved in media and applied to the media surrounding the cell cultures (*n* = 3 for each condition) for 2 h daily for 5 days.

After determining the optimal concentration of metformin, rapamycin, and TRSV, these drugs were applied alone or combined with phytocannabinoids, CBD and THC, and tested on healthy and senescent dermal fibroblasts.

### 4.4. Cell Viability/Cytotoxicity Assays

#### 4.4.1. The Micro-Culture Tetrazolium Assay (MTT)

Cell viability on CCD-1064Sk human neonatal skin fibroblasts was measured by MTT (3-[4,5-dimethylthiazol-2-yl]-2,5-diphenyltetrazolium bromide; thiazolyl blue) colorimetric metabolic activity assay with the cell proliferation kit I (#11465007001, Roche, Mississauga, ON, Canada) according to the manufacturer’s instructions.

Cells were plated at 3.0 × 103 cells/well in 150 μL of cell culture medium in a 96-well assay plate and cultivated for 24–48 h before treatment depending on cell confluency. A broad range of H_2_O_2_ concentrations were examined to determine the appropriate effective/cytotoxic concentration for each designated treatment. Unless otherwise indicated, all measurements were performed in triplicate at specific time points (0, 1, 2, 3, 4, and 5 days). After the desired treatment time, 10 μL of MTT labeling reagent was added to each well without removing media and incubated for 4 h. Afterwards, 100 μL of MTT solubilization solution (10% SDS in 0.01 M HCl) was added to each well, followed by overnight incubation. Cell viability was calculated by comparing it to the control treatment. All experiments were repeated three times (*n* = 3); each test was done in triplicate.

#### 4.4.2. Neutral Red Assay and Microscopy

The neutral red stain is based on the ability of viable cells to incorporate and bind neutral red dye in the lysosomes [91]. It was used to provide a qualitative estimation of the presence of viable cells in the fibroblast cell cultures.

Cells were cultivated in a 24-well cell culture plates and treated appropriately. The medium was removed from the fibroblast cell cultures and the cultures was washed with PBS. After that, 100 μL of Neutral red (Sigma-Aldrich, Saint Louis, MO, USA) dissolved in a cell culture medium (40 μg/mL) was added for each well, followed by a 4 h incubation at the appropriate culture conditions. After incubation, cells were gently washed twice with 150 μL of PBS. Images were taken using a Zeiss Observer Z1 epifluorescence microscope with AxioVision Rel 4.8 software.

### 4.5. RNA Isolation

RNA was isolated from monolayer fibroblast cultures, using TRIzol^®^ Reagent (Invitrogen, Carlsbad, CA, USA); purified using an RNAesy kit (Qiagen, Calgary, AB, Canada), according to the manufacturer’s instructions; and quantified using NanoDrop 2000c (Thermo Fisher Scientific, Wilmington, DE, USA).

### 4.6. Quantitative Real-Time PCR (RT-qPCR)

RT-qPCR was performed on skin fibroblast samples from all experimental groups. According to the manufacturer’s instructions, cDNA was generated with 500 ng RNA using the iScriptTM Select cDNA synthesis kit (Cat# 1708897, BioRad, Hercules, CA, USA). PCR reactions were based on the SsoFastTM EvaGreen^®^ Supermix (Cat# 1725202, BioRad, Hercules, CA, USA) and 500 nM of forward and reverse primers specific for target sequences of interest. Primers were designed using the https://www.idtdna.com/Primerquest (accessed on 1 February 2021) platform (Table S1). Primers were checked before on dilution series of normal fibroblasts cDNA. The reactions were analyzed on a C1000TM Thermo Cycler equipped with a CFX96 Touch™ Real-Time PCR Detection System (BioRad, Hercules, CA, USA). The PCR programs were run according to the SSoFastTM guidelines with annealing temperatures as specified for the specific primer pairs. Expression analysis was performed with the BioRad Software (CFX Manager) and was based on the ΔΔCt method with GAPDH as the housekeeping gene. Each experiment included three biological replicates for each group and two technical replicates per sample.

### 4.7. Wound-Healing Assay

Cells were cultivated to >90% confluence in 24-well plates; 10 µL pipette tips were used to scrape a scratch/wound line through the middle of each well simulating a wound. Cells were washed twice in PBS before adding cell culture growth medium or designated treatments. Images of the healing process were taken on the following time points: 1, 6, 24, 48, and 72 h throughout the assay.

The OLYMPUS CKX41 microscope equipped with an Infinity3 camera was used to collect images within the linear dynamic range representing the range in which the relationship between signal intensity and the amount of material is likely to be linear. Images were analyzed with ImageJ (IJ 1.46r) software. At least seven measurements were counted per sample, and samples were designed in triplicates.

### 4.8. Immunocytochemistry

Cells were plated on glass coverslips for 48 h, treated in 6-well plates, and then fixed in 3% formaldehyde for 20 min at room temperature. Cells were quenched with 50 mM NH_4_Cl in PBS, permeabilized for 5 min in 0.2% Triton X-100 and blocked with 3% BSA for 30 min. After washing, nuclei were stained with 300 nM 4’,6-diamidino-2-phenylindole (DAPI) (Thermo Fisher Scientific, Waltham, MA, USA) in PBS for 15 min before mounting according to the manufacturer’s instructions. Images were taken using a Zeiss Observer Z1 epifluorescence microscope with AxioVision Rel 4.8 software. DAPI produces a blue fluorescence when bound to DNA with excitation at 360 nm and emission at 460 nm. Specimens were stored at 4 °C. Experiments were prepared in triplicates.

### 4.9. QuPath Analysis

QuPath 0.2 was used for quantitative analysis of cell number and stained area for the nuclei of the fibroblasts [113]. Images were taken using a Zeiss Observer Z1 epifluorescence microscope with AxioVision Rel 4.8 software and imported into QuPath. Nuclei were quantified using the cell detection function on the region of interest with parameters optimized to identify nuclei accurately and were confirmed by visual inspection. Any nuclear overlapping or partial nuclei captured erroneously identified as nuclei were deleted. Once nuclei were correctly identified, QuPath provided the following parameters:

Nuclei number—the number of distinct nuclei that were identified

Area of the nucleus (μm^2^)—the number of pixels that enclosed within the nuclear perimeter.

Perimeter of the nucleus (μm)—the number of adjacent pixels in the boundary of the nucleus.

Nucleus circularity—area-to-perimeter ratio which demonstrates the roundness of the nuclear perimeter. This is calculated by multiplying the area by four pi and divided by the square of the convex perimeter. For circular nuclei, the ratio equals 1, whereas nuclei that depart from circularity have ratios less than one.

Nuclear max caliper (μm)—the distance between farthest parallel endpoints touching opposite sides of the nucleus.

Nuclear min caliper (μm)—the distance between closest parallel endpoints touching opposite sides of the nucleus.

Nuclear eccentricity, or ellipticity—the ratio of the min caliper to the max caliper. It shows how oval-shaped the nuclei are.

All data were automatically converted from pixels to the appropriate units and were extracted from QuPath 0.2 software to Microsoft^®^ Office Excel 365 files. Data were imported into the GraphPad Prism software 9.3.1 for statistical analysis (GraphPad Software, San Diego, CA, USA).

### 4.10. Statistical Analysis

The number of cell passages and biological repeats (*n*) for each experiment are indicated in the figure captions. Results are presented as mean of at least three samples per group with standard deviation (SD) of the mean or 95% confidence interval as indicated. Mean values ± SD and statistical analyses were calculated and plotted using GraphPad Prism 9 (GraphPad Software, San Diego, CA) unless stated otherwise. Statistical analysis of data quantification was performed using a one-way ANOVA test (Tukey or Dunnett’s post hoc multiple comparison test). Significance (*p*) was indicated within the figures using the following scale: ** *p* < 0.01; *** p < 0.001; **** *p* < 0.0001.

## 5. Conclusions

In summary, NSRs can act as potential anti-aging compounds but affects skin fibroblasts differently. We found differential effects of NSRs on senescence-associated gene regulation, metabolic and functional maintenance, and cutaneous wound healing. We, for the first time, show that application of TRSV in combination with CBD constitutes a very promising anti-aging and regenerative regimen that can potentially be used for treatment or/and prevention of appearance of aging spots and treating cutaneous wounds. Moreover, we found pCBs alone appeared to be highly efficacious as an anti-aging treatment. Further work should study and test pCBs alone, as well as TRSV in combination with CBD as anti-aging remedies.

## Data Availability

Not applicable.

## Supplementary Materials

The following supporting information can be downloaded at https://www.mdpi.com/article/10.3390/ijms23158804/s1.
