# Supplementary material for: The Effects of Nutrient Signaling Regulators in Combination with Phytocannabinoids on the Senescence-Associated Phenotype in Human Dermal Fibroblasts
Source: Int J Mol Sci. 2022 Aug 8;23(15):8804. doi: 10.3390/ijms23158804 (PMC9368899; doi:10.3390/ijms23158804)
Supplement: Supplementary file 1 [file ijms-23-08804-s001.zip › ijms-1831153-supplementary.pdf]

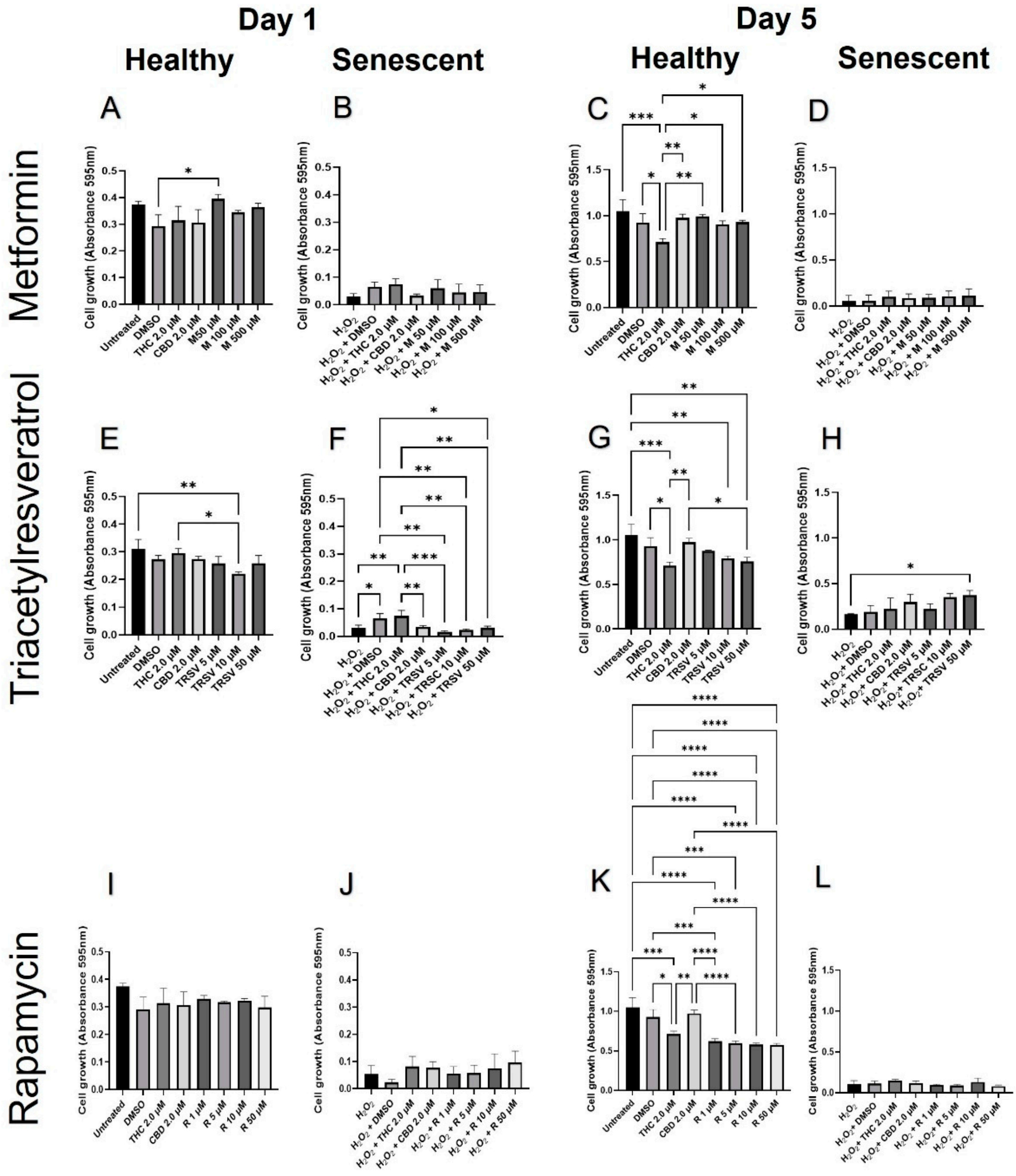

Figure S1. Viability of human skin fibroblasts CCD-1064Sk (p.11) exposed to different concentrations of metformin, triacetylresveratrol and rapamycin compared to THC and CBD. The figure shows changes in cell viability after exposure to NSRs and/or pCBs for: 1 day with metformin in **(A)** healthy and **(B)** senescent cells, 1 day with TRSV in **(C)** healthy and **(D)** senescent cells, 1 day with rapamycin in **(E)** healthy and **(F)** senescent cells, 5 days with metformin in **(G)** healthy and **(H)** senescent cells, 5 days with TRSV in **(I)** healthy and **(J)** senescent cells, 5 days with rapamycin in **(K)** healthy and **(L)** senescent cells. Data were analyzed with a one-way ANOVA test followed by a Tukey post-hoc multiple comparison test. Bars represent mean ± SD. Significance is indicated within the figures using the following scale: **p*<0.05, ***p*<0.01, ****p*<0.001, *****p*<0.0001. CBD, cannabidiol; DMSO, dimethyl sulfoxide (vehicle), M, metformin; R, rapamycin; THC, Δ-9-tetrahydrocannabinol; TRSV, triacetylresveratrol.

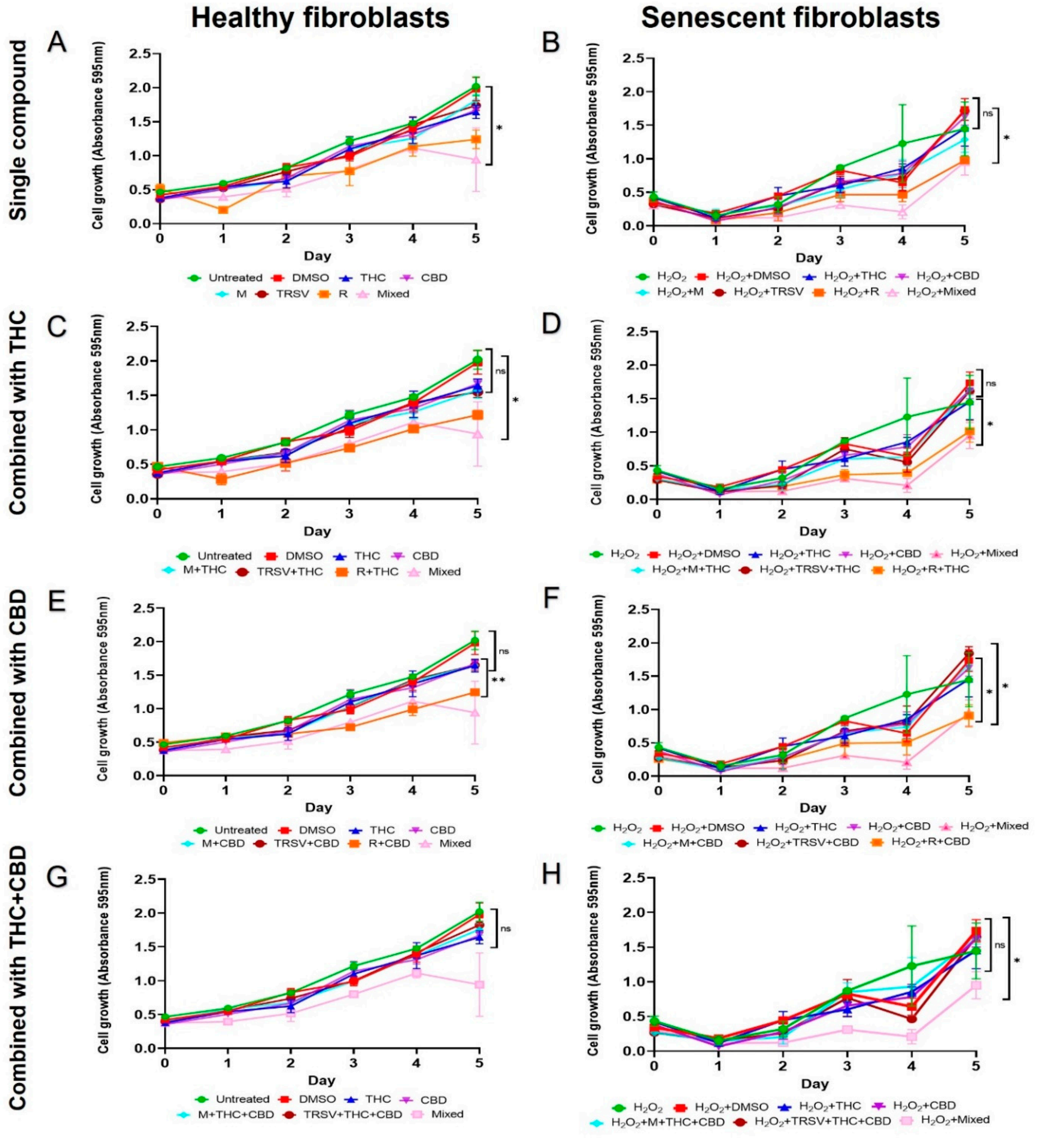

Figure S2. Cell Viability via MTT of dermal fibroblasts CCD-1064Sk (p.11) treated with natural signaling regulators combined with phytocannabinoids. The graphs show cell viability of skin fibroblasts estimated by MTT assay after experimental treatments of **(A,C,E,I)** healthy and **(B,D,F,J)** senescent cells with nutrient signaling regulators combined with THC and CBD. Data were analyzed with a one-way ANOVA test followed by a Tukey post-hoc multiple comparison test. Bars represent mean ± SD. Significance is indicated within the figures using the following scale: **p*<0.05, ***p*<0.01. CBD, cannabidiol; DMSO, dimethyl sulfoxide, M, metformin; Mixed, included metformin + TRSV + rapamycin + THC + CBD; R, rapamycin; THC, Δ-9-tetrahydrocannabinol; TRSV, triacetylresveratrol.

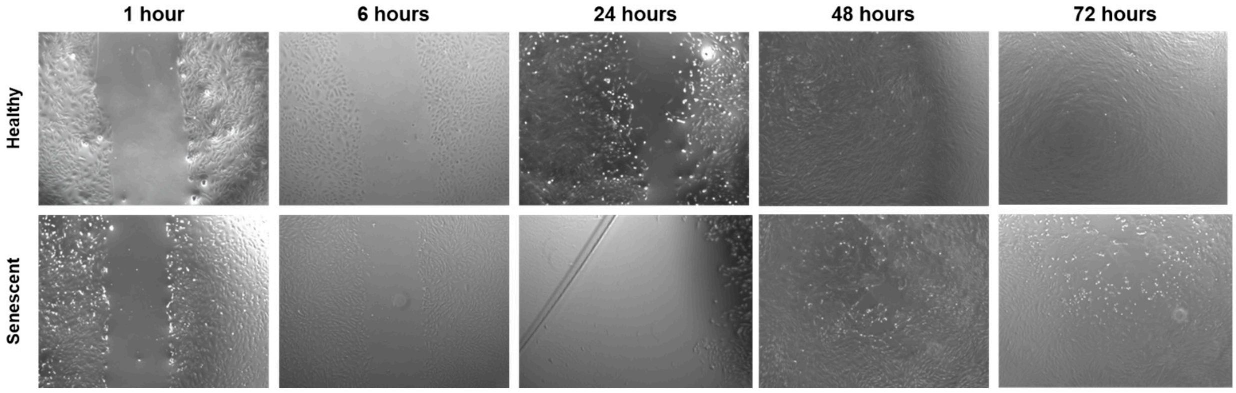

Figure S3. Untreated human skin fibroblasts CCD-1064Sk (p.11) in the wound healing assay. Images have been enlarged from Figure 4 to easily show size of wounds after exposure for 1, 6, 24, 48, and 72 hours in healthy and SIPS fibroblasts.

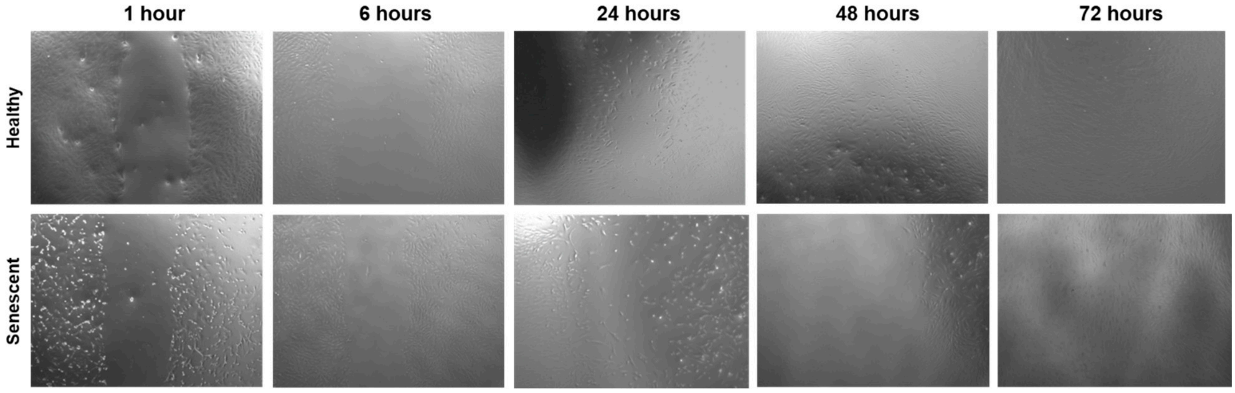

Figure S4. DMSO-treated human skin fibroblasts CCD-1064Sk (p.11) in the wound healing assay. Images have been enlarged from Figure 4 to easily show size of wounds after exposure for 1, 6, 24, 48, and 72 hours in healthy and SIPS fibroblasts.

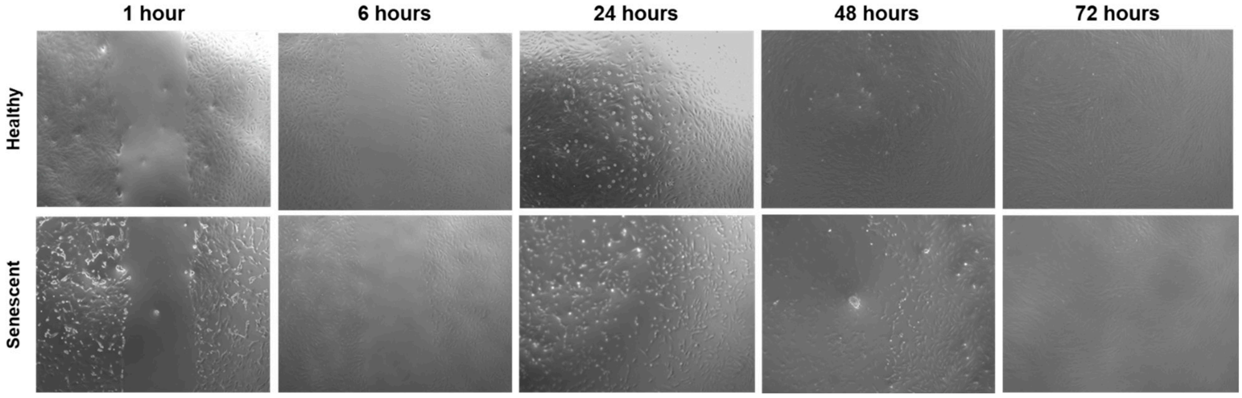

Figure S5. THC-treated human skin fibroblasts CCD-1064Sk (p.11) in the wound healing assay. Images have been enlarged from Figure 4 to easily show size of wounds after exposure for 1, 6, 24, 48, and 72 hours in healthy and SIPS fibroblasts.

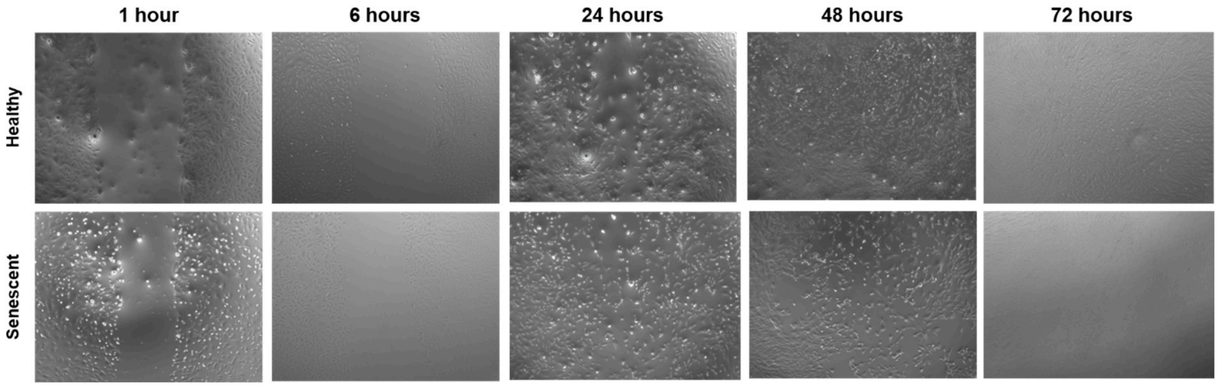

Figure S6. CBD-treated human skin fibroblasts CCD-1064Sk (p.11) in the wound healing assay. Images have been enlarged from Figure 4 to easily show size of wounds after exposure for 1, 6, 24, 48, and 72 hours in healthy and SIPS fibroblasts.

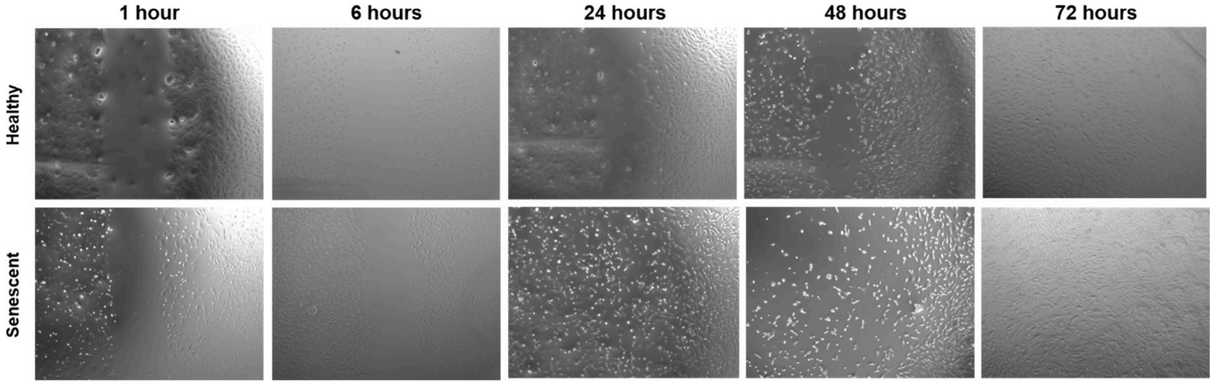

Figure S7. Metformin-treated human skin fibroblasts CCD-1064Sk (p.11) in the wound healing assay. Images have been enlarged from Figure 4 to easily show size of wounds after exposure for 1, 6, 24, 48, and 72 hours in healthy and SIPS fibroblasts.

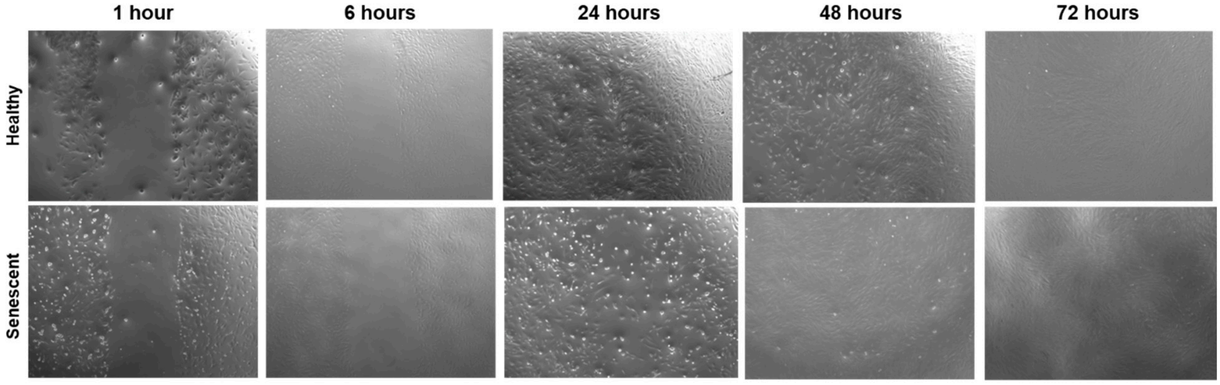

Figure S8. Metformin and THC-treated human skin fibroblasts CCD-1064Sk (p.11) in the wound healing assay. Images have been enlarged from Figure 4 to easily show size of wounds after exposure for 1, 6, 24, 48, and 72 hours in healthy and SIPS fibroblasts.

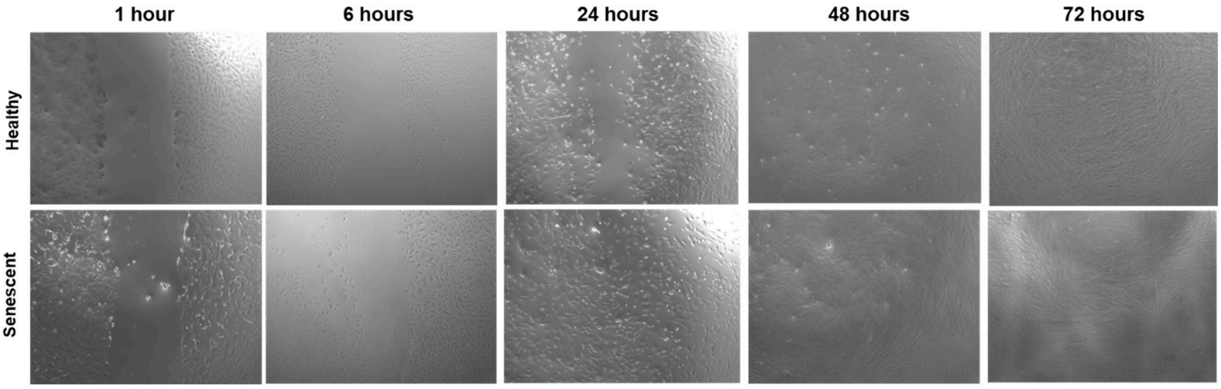

Figure S9. Metformin and CBD-treated human skin fibroblasts CCD-1064Sk (p.11) in the wound healing assay. Images have been enlarged from Figure 4 to easily show size of wounds after exposure for 1, 6, 24, 48, and 72 hours in healthy and SIPS fibroblasts.

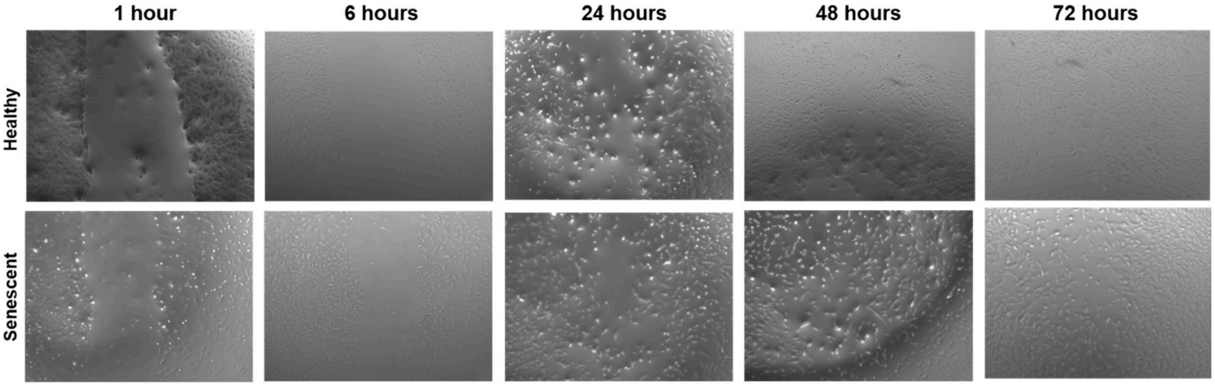

Figure S10. Metformin, THC, and CBD-treated human skin fibroblasts CCD-1064Sk (p.11) in the wound healing assay. Images have been enlarged from Figure 4 to easily show size of wounds after exposure for 1, 6, 24, 48, and 72 hours in healthy and SIPS fibroblasts.

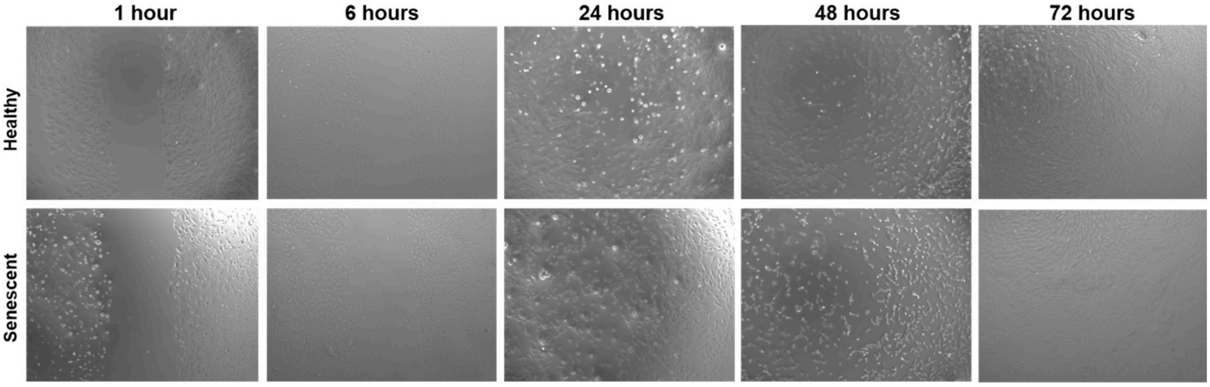

Figure S11. TRSV-treated human skin fibroblasts CCD-1064Sk (p.11) in the wound healing assay. Images have been enlarged from Figure 4 to easily show size of wounds after exposure for 1, 6, 24, 48, and 72 hours in healthy and SIPS fibroblasts.

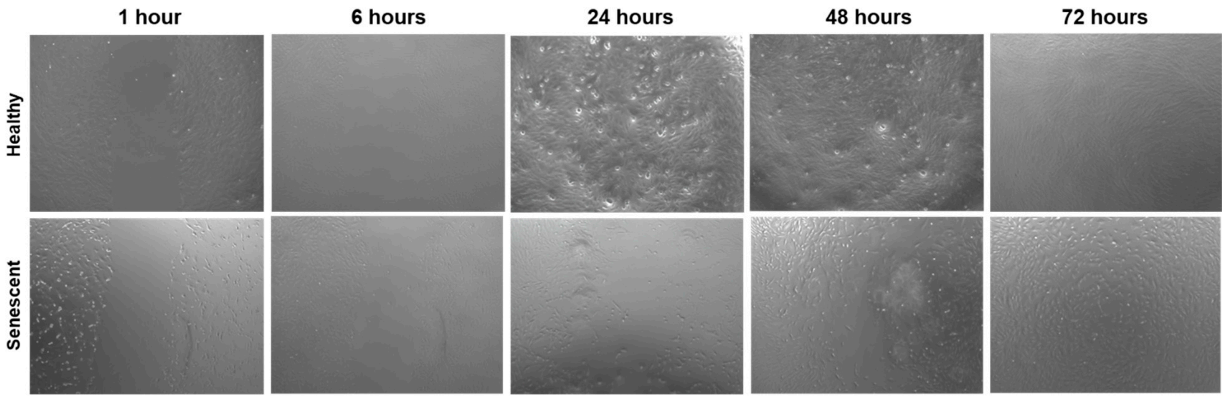

Figure S12. TRSV and THC-treated human skin fibroblasts CCD-1064Sk (p.11) in the wound healing assay. Images have been enlarged from Figure 4 to easily show size of wounds after exposure for 1, 6, 24, 48, and 72 hours in healthy and SIPS fibroblasts.

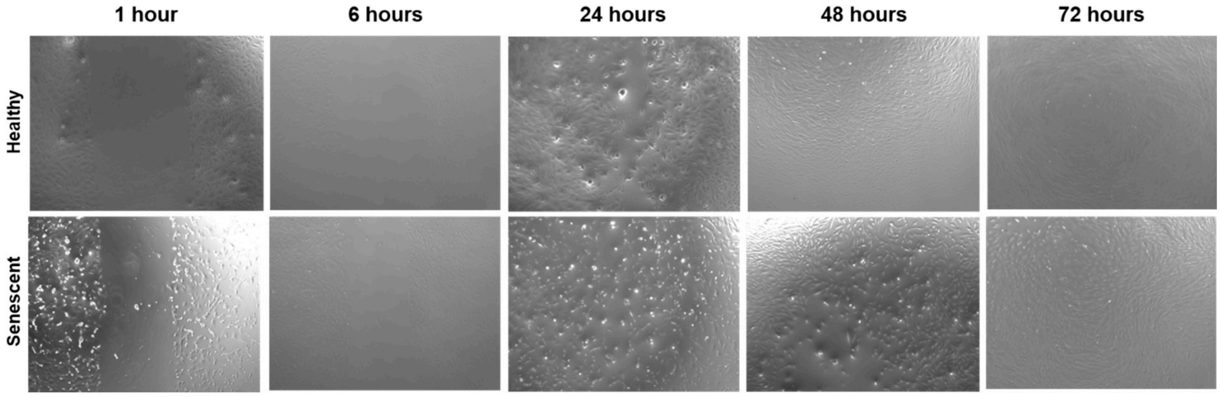

Figure S13. TRSV and CBD-treated human skin fibroblasts CCD-1064Sk (p.11) in the wound healing assay. Images have been enlarged from Figure 4 to easily show size of wounds after exposure for 1, 6, 24, 48, and 72 hours in healthy and SIPS fibroblasts.

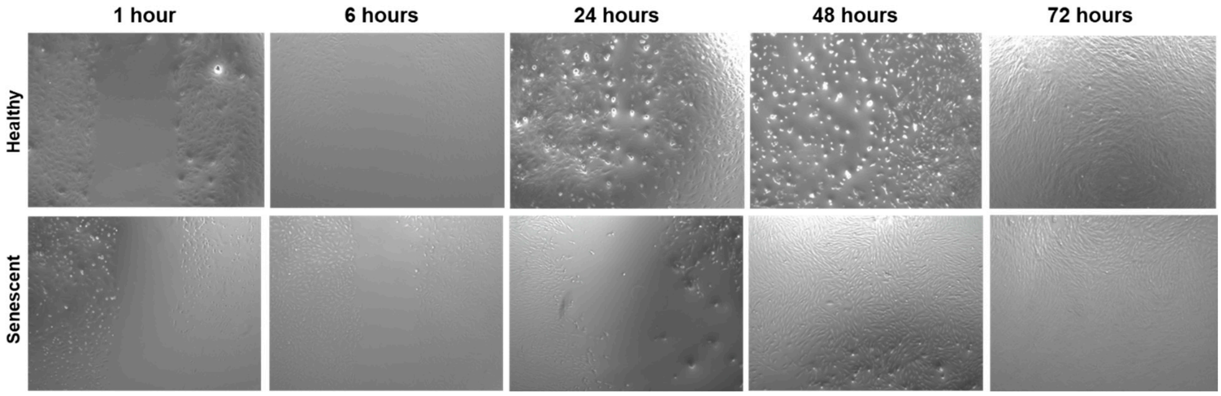

Figure S14. TRSV, THC, and CBD-treated human skin fibroblasts CCD-1064Sk (p.11) in the wound healing assay. Images have been enlarged from Figure 4 to easily show size of wounds after exposure for 1, 6, 24, 48, and 72 hours in healthy and SIPS fibroblasts.

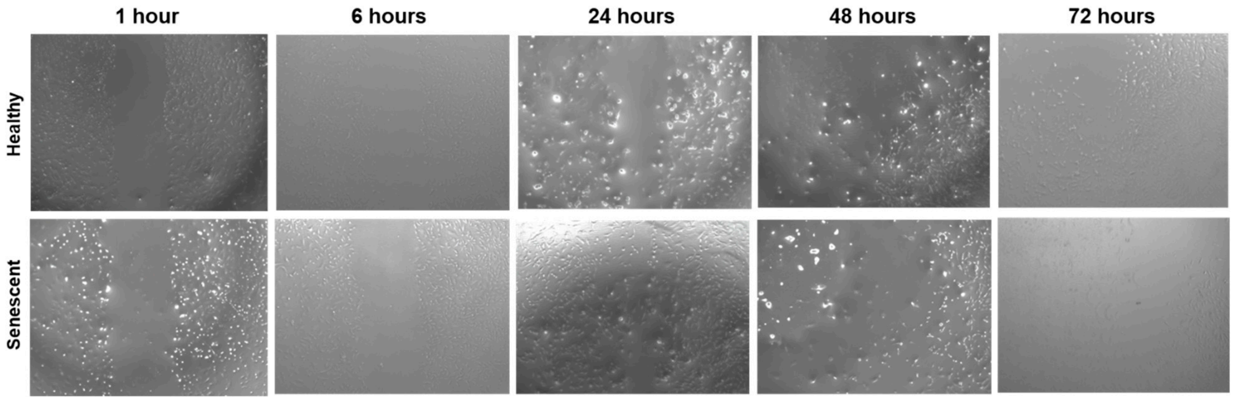

Figure S15. Rapamycin-treated human skin fibroblasts CCD-1064Sk (p.11) in the wound healing assay. Images have been enlarged from Figure 4 to easily show size of wounds after exposure for 1, 6, 24, 48, and 72 hours in healthy and SIPS fibroblasts.

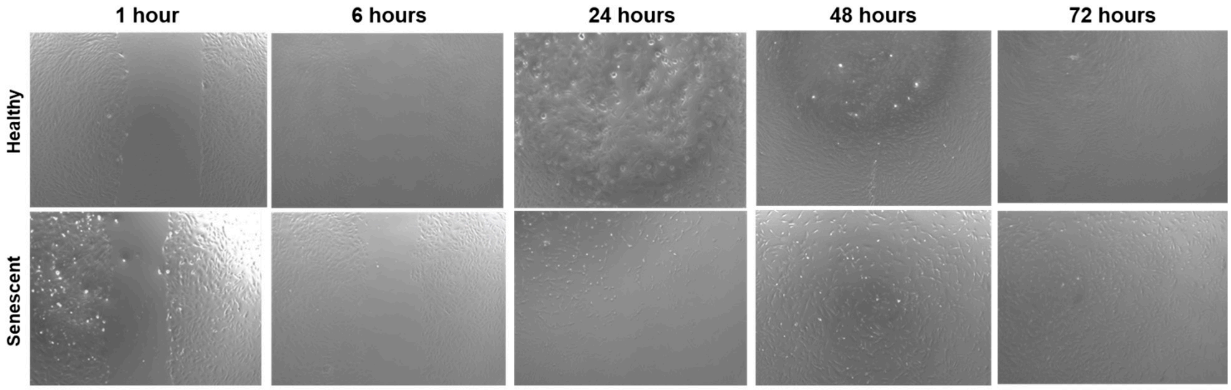

Figure S16. Rapamycin and THC-treated human skin fibroblasts CCD-1064Sk (p.11) in the wound healing assay. Images have been enlarged from Figure 4 to easily show size of wounds after exposure for 1, 6, 24, 48, and 72 hours in healthy and SIPS fibroblasts.

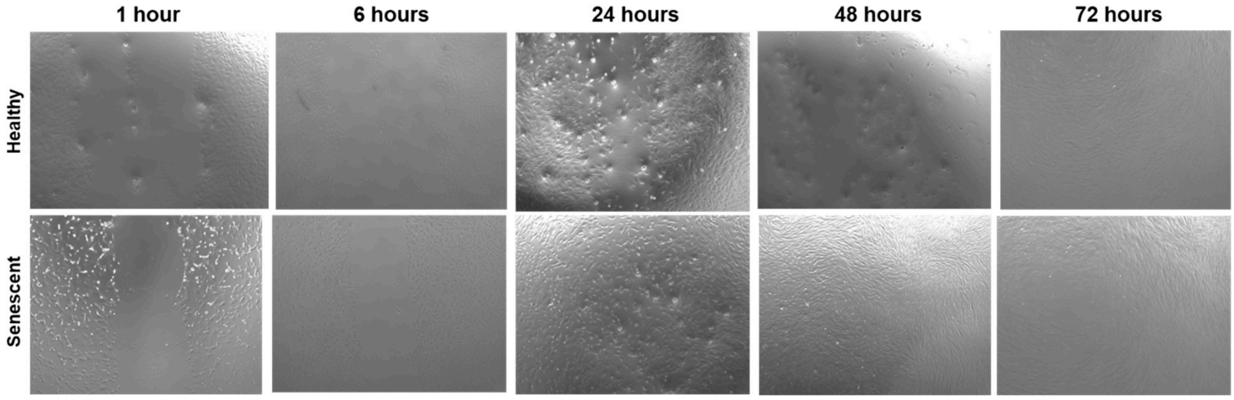

Figure S17. Rapamycin and CBD-treated human skin fibroblasts CCD-1064Sk (p.11) in the wound healing assay. Images have been enlarged from Figure 4 to easily show size of wounds after exposure for 1, 6, 24, 48, and 72 hours in healthy and SIPS fibroblasts.

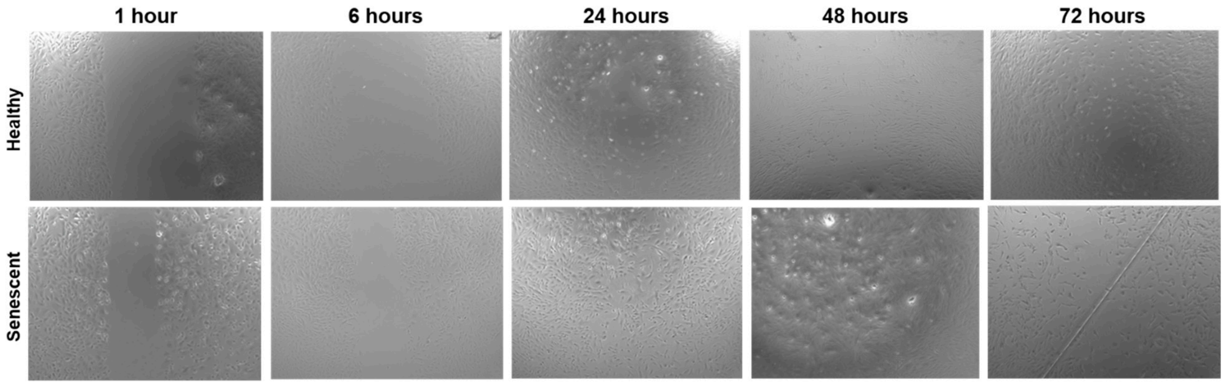

Figure S18. Rapamycin, THC, and CBD-treated human skin fibroblasts CCD-1064Sk (p.11) in the wound healing assay. Images have been enlarged from Figure 4 to easily show size of wounds after exposure for 1, 6, 24, 48, and 72 hours in healthy and SIPS fibroblasts.

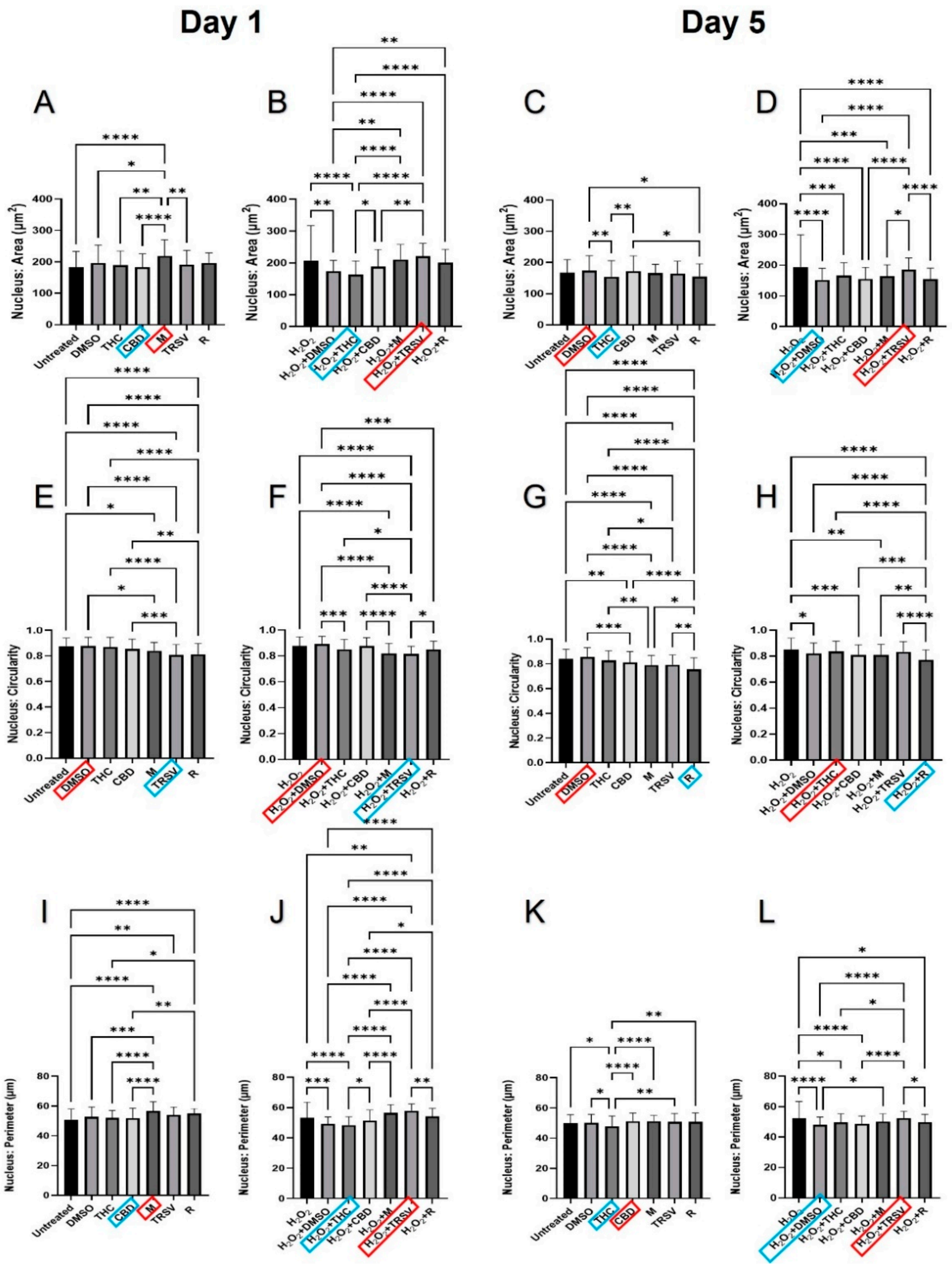

Figure S19. Parameters of DAPI stained nuclei of skin fibroblasts CCD-1064Sk (p.11) exposed to nutrient signaling regulators and phytocannabinoids. **(A,E,I)** nuclear area, circularity and perimeter in healthy fibroblasts treated with 500 μM metformin, 10 μM triacetylresveratrol, 5 μM rapamycin, and 2 μM of pCBs (THC or CBD) for 1 day. **(B,F,J)** nuclear area, circularity and perimeter in senescent fibroblasts after 1 day of treatment. **(C,G,K)** nuclear area, circularity and perimeter in healthy fibroblasts after 5 days of treatment. **(D,H,L)** nuclear area, circularity and perimeter in healthy firboblasts after 5 days of treatment. **(A-D)** nuclear area, **(E-H)** nuclear circularity, **(I-L)** nuclear perimeter. Rectangles depict changes in nuclei parameters: red, highest data; blue, lowest data. Data were analyzed with a one-way ANOVA test followed by a Tukey post-hoc multiple comparison test. Bars represent mean ± SD. Significance is indicated within the figures using the following scale: *p<0.05, **p<0.01, ***p<0.001, ****p<0.0001. CBD, cannabidiol; DMSO, dimethyl sulfoxide (vehicle), M, metformin; R, rapamycin; THC, Δ-9-tetrahydrocannabinol; TRSV, triacetylresveratrol.

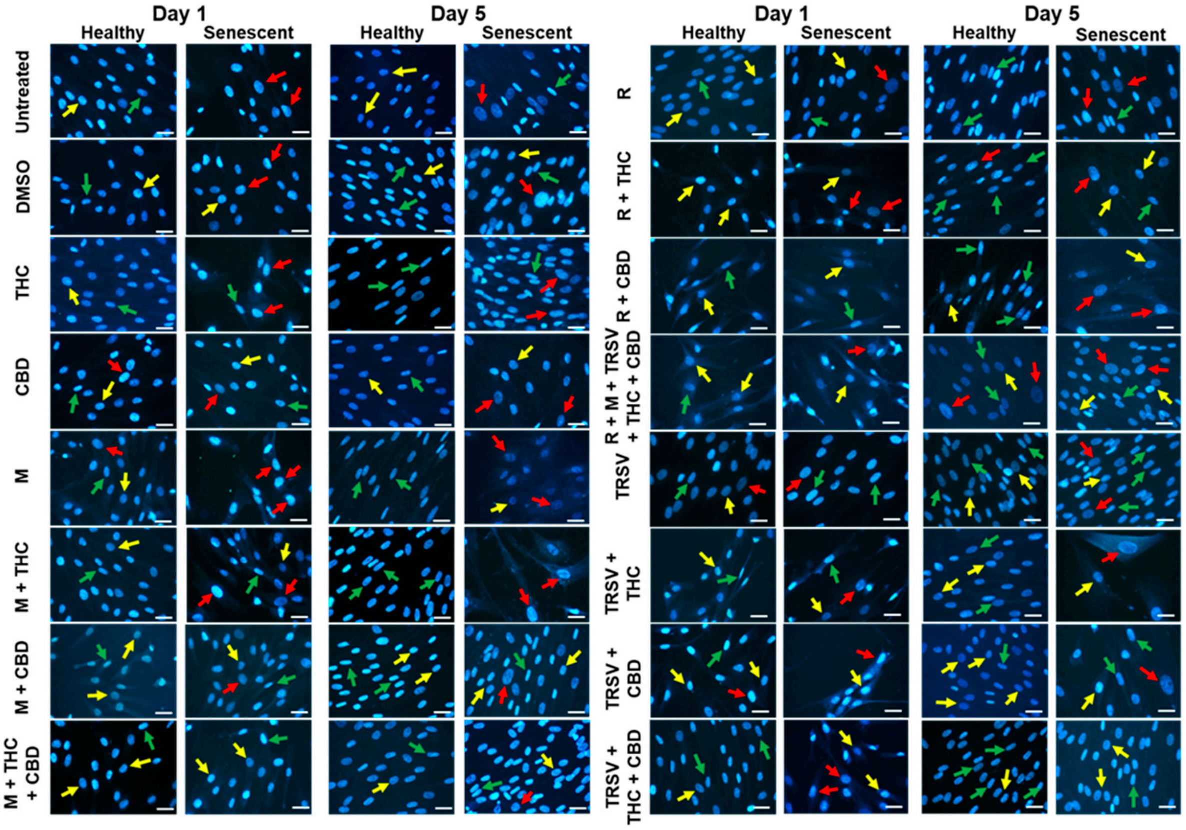

Figure S20. DAPI stained nuclei of dermal fibroblasts CCD-1064Sk (p.11) treated with nutrient signaling regulators and phytoannabinoids. Pictures represent nuclear changes observed by immunofluorescence microscopy in healthy and senescent fibroblasts treated with 500 μM metformin, 5 μM rapamycin, or 10 μM triacetylresveratrol alone or in combination with THC and/or CBD on day 1 and day 5. Arrows depict changes in nuclear shapes: yellow – round, green – elongated, and red – gigantic/irregular. Scale bars = 20 μm. CBD, cannabidiol; DMSO, dimethyl sulfoxide (vehicle), M, metformin; R, rapamycin; THC, Δ-9-tetrahydrocannabinol; TRSV, triacetylresveratrol.

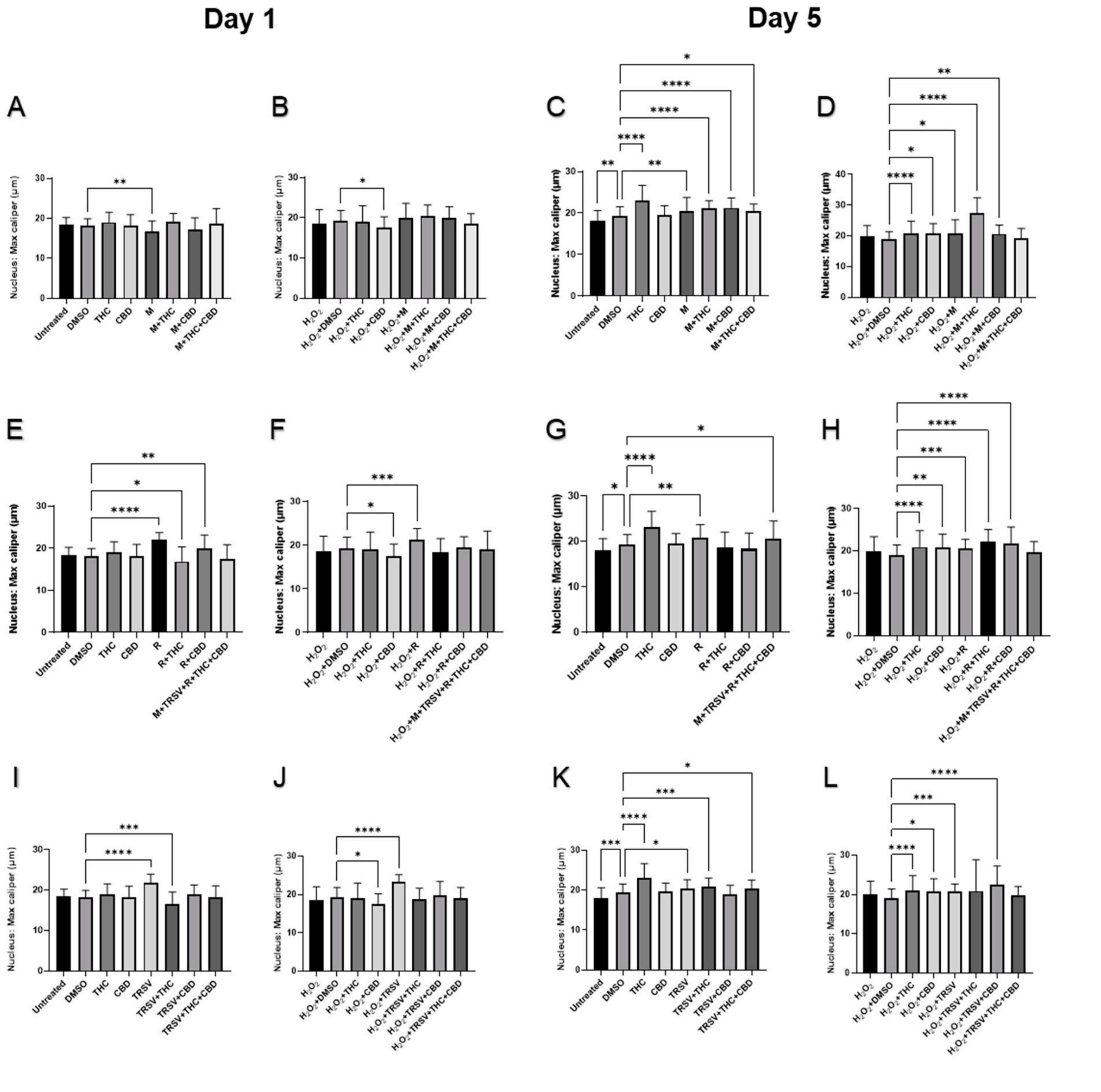

Figure S21. DAPI stained nuclei max caliper parameters of skin fibroblasts CCD-1064Sk (p.11) exposed to metformin, triacetylresveratrol, and rapamycin combined with pCBs. Nuclear parameters were observed by immunofluorescence microscopy for: (A) healthy and (B) senescent fibroblasts treated with 2 µM of pCBs (THC or CBD), 500 µM metformin, and combinations of metformin with THC and/or CBD after 1 day of exposure, (C) healthy and (D) senescent fibroblasts treated with 2 µM of pCBs, 500 µM metformin, and combinations of metformin with THC and/or CBD after 5 days of exposure, (E) healthy and (F) senescent fibroblasts treated with 2 µM of pCBs, 5 µM rapamycin, and combinations of rapamycin with THC and/or CBD after 1 day of exposure, (G) healthy and (H) senescent fibroblasts treated with 2 µM of pCBs, 5 µM rapamycin, and combinations of rapamycin with THC and/or CBD after 1 day of exposure, (I) healthy and (J) senescent fibroblasts treated with 2 µM of pCBs, 10 µM triacetylresveratrol, and combinations of triacetylresveratrol with THC and/or CBD after 5 days of exposure. Data were analyzed with a one-way ANOVA test followed by a Dunnett's post-hoc test compared to the DMSO or H2O2 + DMSO control. Bars represent mean ± SD. Significance is indicated within the figures using the following scale: **p*<0.05, ***p*<0.01, ****p*<0.001, *****p*<0.0001. CBD, cannabidiol; DMSO, dimethyl sulfoxide (vehicle), M, metformin; R, rapamycin; THC, Δ-9-tetrahydrocannabinol; TRSV, triacetylresveratrol.

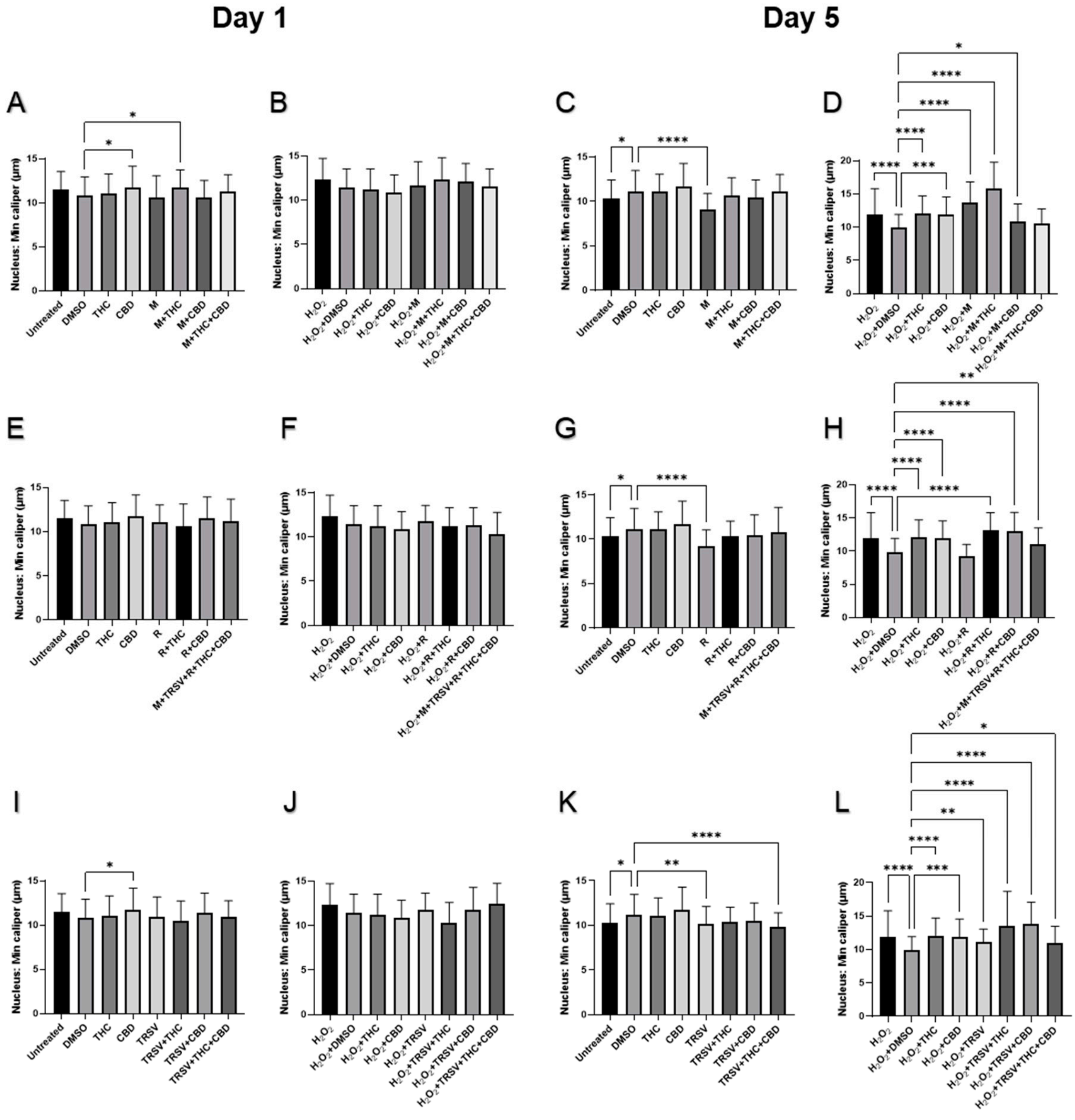

Figure S22. DAPI stained nuclei min caliper parameters of skin fibroblasts CCD-1064Sk (p.11) exposed to metformin, triacetylresveratrol, and rapamycin combined with pCBs. Nuclear parameters were observed by immunofluorescence microscopy for: (A) healthy and (B) senescent fibroblasts treated with 2 µM of pCBs (THC or CBD), 500 µM metformin, and combinations of metformin with THC and/or CBD after 1 day of exposure, (C) healthy and (D) senescent fibroblasts treated with 2 µM of pCBs, 500 µM metformin, and combinations of metformin with THC and/or CBD after 5 days of exposure, (E) healthy and (F) senescent fibroblasts treated with 2 µM of pCBs, 5 µM rapamycin, and combinations of rapamycin with THC and/or CBD after 1 day of exposure, (G) healthy and (H) senescent fibroblasts treated with 2 µM of pCBs, 5 µM rapamycin, and combinations of rapamycin with THC and/or CBD after 1 day of exposure, (I) healthy and (J) senescent fibroblasts treated with 2 µM of pCBs, 10 µM triacetylresveratrol, and combinations of triacetylresveratrol with THC and/or CBD after 5 days of exposure. Data were analyzed with a one-way ANOVA test followed by a Dunnett's post-hoc test compared to the DMSO or H2O2 + DMSO control. Bars represent mean ± SD. Significance is indicated within the figures using the following scale: **p*<0.05, ***p*<0.01, ****p*<0.001, *****p*<0.0001. CBD, cannabidiol; DMSO, dimethyl sulfoxide (vehicle), M, metformin; R, rapamycin; THC, Δ-9-tetrahydrocannabinol; TRSV, triacetylresveratrol.

Table S1. Primer sequences for qPCR analysis

Target Gene	Sequence Forward (5' → 3')	Sequence Reverse (5' → 3')
CNR1 (CB1R)	CAAGCCTCTCTGGCACTTT	CTGGTGGTTGGGCCTATTT
CNR2 (CB2R)	CCTCCCAAAGTGCTAGGATTAC	CTTGTTCTCCTCCCTCATAAGC
COL1A1	CCACGACAAAGCAGAAACATC	GCAACACAGTTACACAAGGAAC
COL3A1	CTGGCATTCCTTCGACTTCT	AGCTTCAGGGCCTTCTTTAC
ELN	CTCAAAGCTGGATTCGCTCTA	AAGGGCAAGGTGGCTATTC
MMP2	AGAGAACCTCAGGGAGAGTAAG	CCTCGAACAGATGCCACAATA
CDKN2A (P16)	AGCTGTCGACTTCATGACAAG	GAGCTTTGGTTCTGCCATTTG
CDKN1A (P21)	CCTTCCAGCTCCTGTAACATAC	TCGAGAGGTTTACAGTCTAGGT
SIRT1	AGAACCCATGGAGGATGAAAG	TCATCTCCATCAGTCCCAAATC
SIRT6	CCTCTGACTTGCTGTGTTGT	GAGGGAGTTCACTCCTGTTTAAG
TP53	AGGGATGTTTGGGAGATGTAAG	CCTGGTTAGTACGGTGAAGTG
NFKB1	GAGACATCCTTCCGCAAACT	GGTCCTTCCTGCCCATAATC
GAPDH	CAGGAGGCATTGCTGATGAT	GAAGGCTGGGGCTCATTT
